# Combined Use of a Solid-Phase Hexapeptide Ligand Library with Liquid Chromatography and Two-Dimensional Difference Gel Electrophoresis for Intact Plasma Proteomics

**DOI:** 10.1155/2011/739615

**Published:** 2011-09-08

**Authors:** Tatsuo Hagiwara, Yumi Saito, Yukiko Nakamura, Takeshi Tomonaga, Yasufumi Murakami, Tadashi Kondo

**Affiliations:** ^1^Division of Pharmacoproteomics, National Cancer Center Research Institute, Chuo-ku, Tokyo 104-0045, Japan; ^2^Laboratory of Genome Biology, Department of Biological Science and Technology, Tokyo University of Science, Tokyo 278-8510, Japan; ^3^Laboratory of Proteome Research, National Institute of Biomedical Innovation, Osaka 567-0085, Japan

## Abstract

The intact plasma proteome is of great interest in biomarker studies because intact proteins reflect posttranslational protein processing such as phosphorylation that may correspond to disease status. We examined the utility of a solid-phase hexapeptide ligand library in combination with conventional plasma proteomics modalities for comprehensive profiling of intact plasma proteins. Plasma proteins were sequentially fractionated using depletion columns for albumin and immunoglobulin, and separated using an anion-exchange column. Proteins in each fraction were treated with a solid-phase hexapeptide ligand library and compared to those without treatment. Two-dimensional difference gel electrophoresis demonstrated an increased number of protein spots in the treated samples. Mass spectrometric studies of these protein spots with unique intensity in the treated samples resulted in the identification of high- and medium-abundance proteins. Our results demonstrated the possible utility of a solid-phase hexapeptide ligand library to reveal greater number of intact plasma proteins. The characteristics of proteins with unique affinity to the library remain to be clarified by more extensive mass spectrometric protein identification, and optimized protocols should be established for large-scale plasma biomarker studies.

## 1. Introduction

The plasma proteome has been extensively investigated with the aim of biomarker development [1, 2]. Plasma is the most accessible clinical material, and plasma biomarkers for early diagnosis and monitoring the response to therapy and disease recurrence would be beneficial for patients with cancer. Because proteins released by tumors, particularly early-stage tumors, are expected to exist in very low concentrations and plasma contains various proteins with considerable heterogeneity between and within patients, the identification of novel plasma biomarkers represents a substantial challenge. 

Global expression studies on intact plasma proteins are of special interest in biomarker studies as the intact proteins reflect the functional features of protein structure. Those include posttranslational processing such as phosphorylation and glycosylation. Peptide subsets from complex digests have been analyzed for plasma proteomics, resulting in the identification of low-abundance proteins such as tissue leakage proteins [3] and biomarker candidates [4]. However, analysis of peptide digests may not be sensitive to posttranslational protein processing, and may therefore not reveal many relevant protein isoforms associated with disease status. To date, much effort has been devoted to detect trace intact proteins in complex plasma samples.

The utility of a combinatorial hexapeptide ligand library immobilized on a solid-phase matrix has been reported, introduced to intact plasma proteomics [5–9], and commercialized as ProteoMiner (Bio-Rad Laboratories, Hercules, CA, USA). ProteoMiner contains millions of randomly synthesized hexapeptide ligands that are equally represented with a selected number of targets. When a complex plasma protein extract is exposed, the hexapeptide ligands for high-abundance proteins are saturated, but the majority remains unbound. In contrast, the proteins which do not saturate the corresponding hexapeptide ligands and usually not observed by the conventional methods will appear in the proteome data. The approach of using a combinatorial hexapeptide ligand library is different from that of using depletion and separation; thus, it reveals a novel aspect of the plasma proteome. A recent report demonstrated that prefractionation using a hexapeptide ligand library for shotgun mass spectrometry studies identified plasma proteins not recorded in the Human Plasma Proteome Project [10]. The combined use of a hexapeptide ligand library with depletion and separation methods has also been a challenge in deeper plasma proteomics [11], and the resulting protein contents are examined by gel electrophoresis and mass spectrometry [12, 13]. ProteoMiner has been used for disease biomarker studies in lung cancer [14] and liver cancer [15]. Considering that it will potentially visualize the unique plasma proteome aspects, the application and optimization of a solid-phase hexapeptide ligand library for disease biomarker studies should be further investigated. 

In this study, we examined the utility of a solid-phase hexapeptide ligand library in combination with a depletion column, an anion-exchange column, and 2D-DIGE that allows an instant visual comparison of the protein patterns. Protein spots exhibiting prominent differences between samples treated with and without the library were subjected to mass spectrometry. Our study clearly demonstrated that the combined use of the ProteoMiner and the other proteomics modalities can visualize unique plasma proteome.

## 2. Materials and Methods

### 2.1. Sample Preparation

Frozen human plasma was purchased from Cosmobio KOJ (Tokyo, Japan). After the plasma was placed on ice, 40 mL plasma was centrifuged and 30 mL supernatant was recovered for the following experiments.

### 2.2. Albumin Depletion

Albumin and other proteins were separated using a HiTrap Blue HP column (5 mL resin, GE, Uppsala, Sweden) with the AKTA Explorer system (GE) at a flow rate of 1.0 mL/min. The separation was initiated by washing the column with rinse buffer (50 mM KH_2_PO_4_/Na_2_HPO_4_, pH 7.0) for 5 min. Plasma (30 mL) was diluted with 60 mL 50 mM KH_2_PO_4_/Na_2_HPO_4_ (pH 7.0), and 9 mL of the diluted plasma was injected. The column was then washed with binding buffer (50 mM KH_2_PO_4_/Na_2_HPO_4_, pH 7.0) for 35 min, and the flow-through fraction was collected. Bound proteins were eluted from the column with elution buffer (50 mM KHPO_4_, 1.5 M KCl, pH 7.0) for 45 min, and the bound fraction was collected. The column was neutralized with rinse buffer for 20 min. This process was repeated 10 times for a total of 90 mL of diluted plasma. 

One-third of the flow-through and bound fractions, approximately 150 mL of each, was concentrated to 1.2 mL using a VIVA Spin 20 column (10 K MWCO, 20 mL capacity, Sartorius, Gotteingen, Germany). Then, 1.0 mL and 0.20 mL of the concentrated samples were subjected to treatment with the solid-phase hexapeptide ligand library and 2D-DIGE, respectively. Two-thirds of the flow-through fraction, approximately 300 mL, was subjected to an immunodepletion column.

### 2.3. Immunoglobulin Depletion

Immunoglobulin was depleted using the HiTrap Protein G HP column (1 mL resin, GE) with the AKTA Explorer system (GE) at a flow rate of 1.0 mL/min. The depletion was initiated by washing the column with rinse buffer (50 mM KH_2_PO_4_/Na_2_HPO_4_, pH 7.0) for 4 min. After 15 mL of the flow-through fraction from the HiTrap Blue HP column was injected, the column was washed with binding buffer (50 mM KH_2_PO_4_/Na_2_HPO_4_, pH 7.0) for 5 min, and the flow-through fraction was collected. Bound proteins were eluted from the column with elution buffer (0.1 M glycine-HCl, pH 2.2) for 8 min and collected as the bound fraction. The collected bound fraction was immediately neutralized with neutralizing buffer (1.0 M Tris-HCl, pH 9.0). The column was equilibrated with rinse buffer for 5 min for reuse. This process was repeated 20 times for a total of two-thirds of the flow-through fraction from the HiTrap Blue HP column (approximately 300 mL). 

Half of the flow-through and bound fractions (approximately 200 mL and 80 mL, resp.) were concentrated to 1.2 mL and 0.25 mL, respectively, using VIVA Spin 20 columns (Sartorius). Then, 1.0 mL of the concentrated flow-through fraction and 0.20 mL of the concentrated bound fraction were subjected to treatment with the solid-phase ligand library, and the remaining samples were subjected to 2D-DIGE. Another half of the flow-through fraction (approximately 200 mL) was concentrated to 2.0 mL using the VIVA Spin 20 column (Sartorius). After diluting with 38 mL of 25 mM Tris-HCl (pH 9.0), the sample was subjected to separation on an anion-exchange column.

### 2.4. Anion Exchange

The flow-through fraction from the HiTrap Protein G HP column was separated using the Resource Q column (1 mL resin, 6.4 mm id × 30 mm, GE) with the AKTA Explorer system (GE) at a flow rate of 3.0 mL/min. The separation was initiated by washing the column with rinse buffer (25 mM Tris-HCl, pH 9.0) for 4 min, and 5 mL of the flow-through fraction from the HiTrap Protein G HP column was injected. The separations were performed using a stepwise NaCl gradient as follows: 0, 100, 150, 200, 250, and 1000 mM for 5 min each. All samples contained 25 mM Tris-HCl, pH 9.0. The column was washed with rinse buffer (25 mM Tris-HCl, pH 9.0) for 5 min. This process was repeated 8 times for a total of 40 mL of the diluted flow-through fraction from the HiTrap Protein G HP column. 

The collected samples were concentrated to 0.25 mL, and the buffer was exchanged gradually with 25 mM Tris-HCl (pH 9.0) using the VIVA Spin 20 column (Sartorius). Then, 0.2 mL and 0.05 mL were subjected to treatment with the solid-phase ligand library and 2D-DIGE, respectively.

### 2.5. Treatment with the Solid-Phase Ligand Library

A solid-phase combinatorial library of hexapeptides was purchased from Bio-Rad Laboratories (ProteoMiner kit). Unprocessed plasma (1 mL) and the flow-through fractions from the HiTrap Blue HP and HiTrap Protein G HP columns were treated using the ProteoMiner large-capacity kit, and 0.2 mL of the bound fraction from the HiTrap Protein G HP column, and all fractions from the Resource Q column were treated using the ProteoMiner small-capacity kit. After 2 h of incubation at room temperature, the unbound fraction was washed out by centrifugation. After rinsing, the bound sample was eluted with an elution reagent containing 8 M urea, 2% CHAPS, and 5% acetic acid, according to the manufacturer's instructions.

### 2.6. Measurement of Protein Concentration

Protein concentration was measured using a protein assay kit (Bio-Rad), according to the manufacturer's instructions (Table 1).

### 2.7. SDS-PAGE

Protein samples (1 *μ*g) were examined by electrophoresis using 18-well precast 12.5% polyacrylamide gel plates (e-PAGEL, ATTO, Tokyo, Japan). Electrophoresis was performed at a constant current of 40 mA for 80 min and using the page Run AE6531 system [16]. Silver staining was performed using the Silver Stain KANTO III kit (Kanto Chemical, Tokyo, Japan), according to the manufacturer's instructions. 

### 2.8. 2D-DIGE

2D-DIGE was performed as described previously [17]. Briefly, protein samples (20 *μ*g) were labeled with the Cy3 or Cy5 fluorescent dye (CyDye DIGE Fluor saturation dye, GE), and differentially labeled protein samples were mixed. After dividing into 3, the labeled protein samples were separated by 2D-PAGE. The first-dimension separation was performed using a 24 cm length immobiline gel (IPG, pI 4–7, GE) and Multiphor II (GE) whereas the second-dimension separation was performed using gradient gels prepared in house and EttanDalttwelve (GE). The gels were scanned using a laser scanner (Typhoon Trio, GE) at an appropriate wavelength for Cy3 or Cy5. The Cy3 and Cy5 intensities were compared in the same gel using the Progenesis SameSpots software (version 4.0; Nonlinear Dynamics, Newcastle, UK). ProteoMiner-treated and untreated samples were labeled with Cy3 and Cy5, respectively, or with Cy5 and Cy3, respectively. Six gels were run for each sample. The average value of the intensity ratio was calculated among the triplicate gels for all protein spots and then averaged between the 2 samples for further study. Spot intensity data were exported from the Progenesis SameSpots software as Excel files amenable to numerical data analysis. 

### 2.9. Mass Spectrometric Protein Identification

Proteins were extracted from the protein spots by in-gel digestion, as reported previously [17]. Briefly, protein samples (100 *μ*g) were labeled with Cy3 and separated by 2D-PAGE. The protein spots were then recovered from the gel pieces using an automated spot recovery machine. The recovered protein spots were extensively washed with a solution containing acetonitrile and ammonium bicarbonate minimum and treated with trypsin (Promega, Madison, WI, USA) at 37°C overnight. The tryptic digests were recovered from the gel pieces, concentrated by vacuum, and resolubilized with 0.1% trifluoroacetic acid. The final tryptic digests were subjected to mass spectrometry, which was performed using the LXQ linear ion trap mass spectrometer (Thermo Electron, San Jose, CA, USA). The Mascot software (version 2.3.0; Matrix Science, London, UK) was used to search for the mass of the peptide ion peaks against the SWISS-PROT database (Homo sapiens, 471472 sequences in Sprot_57.5 fasta file). The search parameters were as follows: trypsin digestion allowing up to 3 missed tryptic cleavages, fixed modifications of carbamidomethyl, variable modifications of oxidation, 1^+^, 2^+^, and 3^+^ peptide charge, peptide mass tolerance of 2.0 Da, and use of MS/MS tolerance of 1.0 Da for all tryptic-mass searches.

## 3. Results and Discussion

We previously reported the utility of combining multidimensional chromatography and 2D-DIGE for intact plasma proteomics. Extensive fractionation by the different separation modes increased the number of protein spots on 2D-DIGE and allowed a quantitative comparison between the plasma samples from healthy donors and those from patients with lung adenocarcinoma [18] and pancreatic cancer [19]. However, mass spectrometric protein identification revealed that protein spots with a significant difference between the sample groups corresponded to high- and medium-abundance proteins such as acute-phase proteins, but no known plasma tumor markers were detected. Thus, we concluded that further investigations are needed to reveal low-abundance proteins for biomarker studies. In this study, we examined whether a novel technology, a solid-phase hexapeptide ligand library could improve the linkage of multidimensional chromatography and 2D-DIGE. 

### 3.1. Overall View of Protein Fractionation: Comparison and Detection

**Figure 1:**
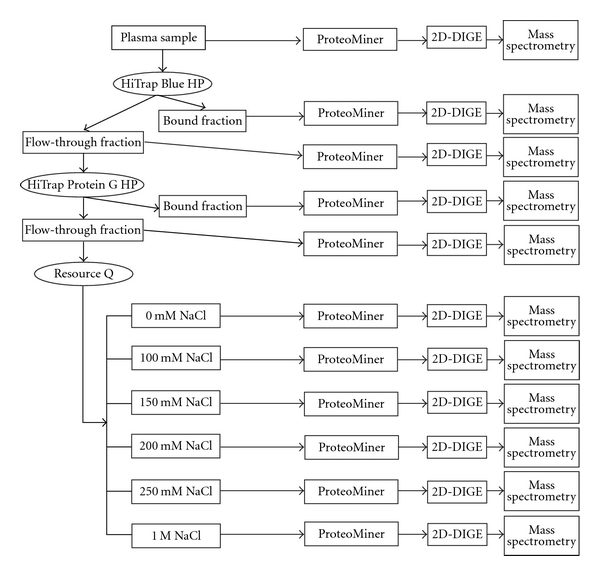
Overview of protein fractionation by sequential use of 3 different columns to separate plasma proteins. All fractions were subjected to ProteoMiner and 2D-DIGE.

The overall view of sequential protein separation is shown in Figure 1. A sample equivalent to 10 mL plasma was separated using 3 different columns and then treated with the solid-phase hexapeptide ligand library ProteoMiner. The ProteoMiner-treated and untreated samples were compared using 2D-DIGE by labeling them with different fluorescent dyes and separating the labeled proteins on an identical gel. The protein spots with significantly different intensities between the ProteoMiner-treated and untreated samples were subjected to mass spectrometry to identify the proteins. 

The number of observable low-abundance proteins was affected by the initial amount of plasma sample and the sensitivity of the final quantification method. We used a relatively large volume of plasma sample (10 mL) as the initial material. The immunodepletion columns allow the use of only a small volume of plasma sample for separation. Furthermore, a significantly larger number of plasma samples should be examined to obtain conclusive results for biomarker development. A larger volume of samples can be manipulated by repeatedly using the same immunodepletion column. Although it is quite feasible, special attention may be required to maintain reproducibility during a long period of use. In this study, we used Blue Sepharose and Protein G-Sepharose columns in a sequential manner to deplete albumin and subsequently immunoglobulin and to minimize repeated use of the same column. Although these columns may have less sensitivity than an immunodepletion column and deplete nontargeted proteins that may bind to albumin and immunoglobulin, a larger volume of plasma sample can be treated in individual procedures. A previous study indicated that Cibacron Blue beads remove a major portion of the albumin but with concomitant loss of potentially important peptides and proteins [20]. Thus, we examined both the column-bound and flow-through fractions (Figure 1). Although the specificity of Cibacron Blue beads was not validated in this study, as the purpose of Cibacron Blue was to reduce the complexity of plasma sample, it should not be problem. 

To avoid possible redundant proteins in the neighboring fractions as much as possible when utilizing the anion-exchange column, we used stepwise elution and fractionation; once all proteins were eluted, the next elution buffer was applied to the column (Figure 1). Considering the complexity of the samples and resolution of an anion-exchange column, extensive fractionation with a gradient buffer system may result in redundant contents among the fractions. We employed 6 stepwise fractionations by monitoring the fraction contents using SDS-PAGE (data not shown).

### 3.2. High Reproducibility of Protein Fractionation by Chromatography

The ultraviolet detection (280 nm) trace for each run demonstrated consistent separation of albumin and immunoglobulin from the depletion and anion-exchange columns. This high reproducibility may suggest the possible utilities of this approach for biomarker studies (Supplementary Figure 1). High quantitative and qualitative reproducibility of the solid-phase hexapeptide ligand library ProteoMiner has been confirmed in previous reports [21, 22]. 

### 3.3. Demonstration of the Effects of Fractionation and Dynamic Range Reduction

**Figure 2:**
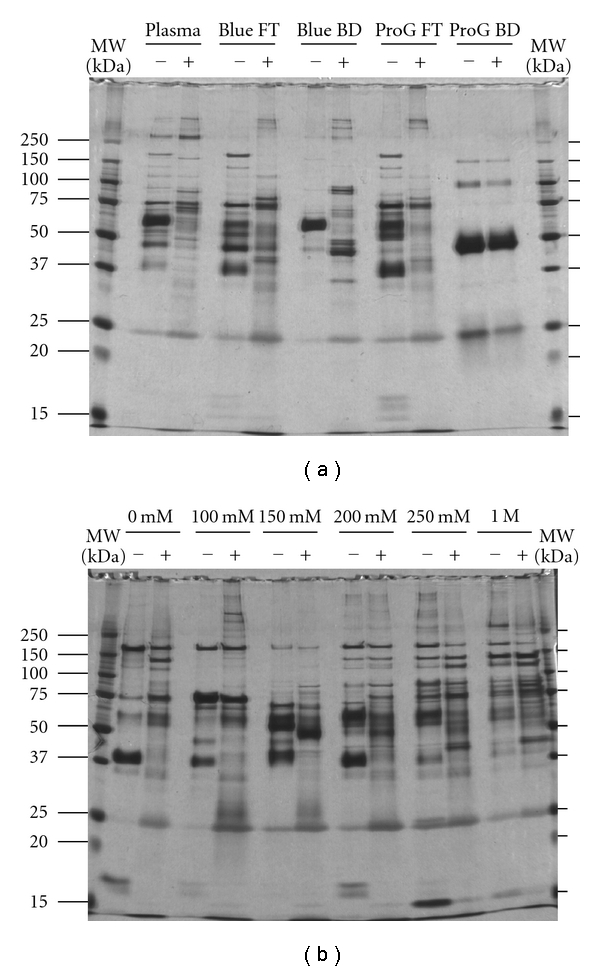
Overview of the protein contents fractionated by liquid chromatography and ProteoMiner. The fractionated protein samples were loaded onto SDS-PAGE, and the protein contents were visualized by silver staining.

We examined the effects of sequential plasma protein fractionation using 3 columns and the reduction of dynamic range by ProteoMiner (Figure 2). The contents of the fractionated samples were apparently different from each other. Notably, the protein sample bound to the Blue Sepharose and Protein G-Sepharose columns included many proteins that should be different from the targeted proteins, according to their molecular weights. Treatment of the fractionated samples with ProteoMiner enhanced the proteins that were not observed, except for those bound to the Protein G-Sepharose column. Treatment of the bound fraction from the Protein G-Sepharose column with ProteoMiner did not result in a greater number of observable proteins. This may have been due to the low complexity and narrow dynamic range of proteins in the bound fraction from the Protein G-Sepharose column. There was one order of magnitude in concentration difference for the observed protein bands in the ProteoMiner-treated sample. These observations may reflect that the affinity of proteins for the peptide may not be equal and even the number of peptides bound on the beads is equal, the amount of proteins bound to the ProteoMiner may be different depending on their affinity. This fraction contained similar amounts of only 4 major proteins, as revealed by SDS-PAGE (Figure 2), and they may have been absorbed to ProteoMiner in proportion to their original amount. 

The concentration and amount of protein samples before and after ProteoMiner treatment are summarized in Supplementary Table  1. The recovery rate from ProteoMiner was between 0.54 and 6.33%, suggesting that a unique population of protein species selectively bound to ProteoMiner. This assumption was supported by the SDS-PAGE data, except the bound fraction from the Protein G-Sepharose column which included only 4 major proteins that were bound to ProteoMiner (Figure 2).

### 3.4. Higher Separation of Fractionated Protein Samples and an Evaluation of the Effects of ProteoMiner Treatment

Although SDS-PAGE separated individual proteins with higher resolution than chromatography in this study, using it for a quantitative comparison in a biomarker study may be troublesome because many protein bands obviously overlapped (Figure 2). Thus, we subjected the fractionated samples to 2D-DIGE in order to separate the proteins with higher resolution. Bandow compared ProteoMiner-treated and untreated plasma samples using conventional 2D-PAGE and demonstrated substantial differences between unprocessed and immunodepleted plasma samples [11]. In 2D-DIGE, 2 protein samples were labeled with different fluorescent dyes, mixed, and separated by 2D-PAGE. Because the 2 samples were separated on an identical 2D-PAGE, gel-to-gel variation was compensated. In addition, the wide dynamic range of the fluorescent dyes enabled a quantitative comparison. 2D-DIGE has been applied to compare the performance of ProteoMiner with an immunodepletion column [12]. We further extended the evaluation of the utility of ProteoMiner by loading a high amount of protein and examining the proteins separated by an anion-exchange column. 

**Figure 3:**
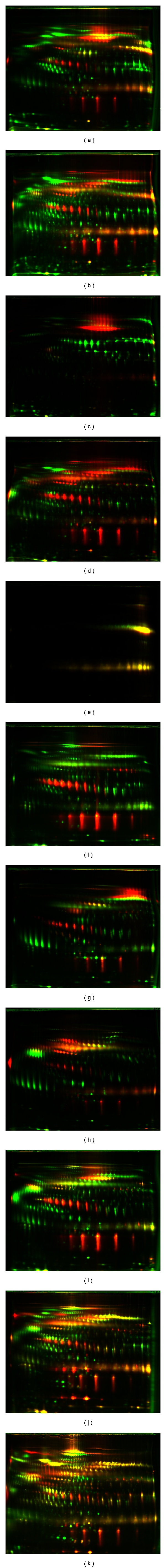
Effects of ProteoMiner treatment were examined by 2D-DIGE. The ProteoMiner-treated and untreated samples were labeled with Cy3 and Cy5, respectively, mixed, and separated by 2D gel electrophoresis. Note that a significant number of protein spots showed different intensities between the 2 samples. The dye-swapped images are shown in Supplementary Figure 2. (a) Original plasma; (b) flow-through fraction of HiTrap Blue HP column; (c) binding fraction of HiTrap Blue HP column. (d) Flow-through fraction of HiTrap Protein G HP column. (e) Binding fraction of HiTrap Protein G HP column; 0 mM fraction. (f) 100 mM fraction. (g) 150 mM. (h) 200 mM. (i) 250 mM. (j) 1 M fraction. (k) Resource Q column.

**Figure 4:**
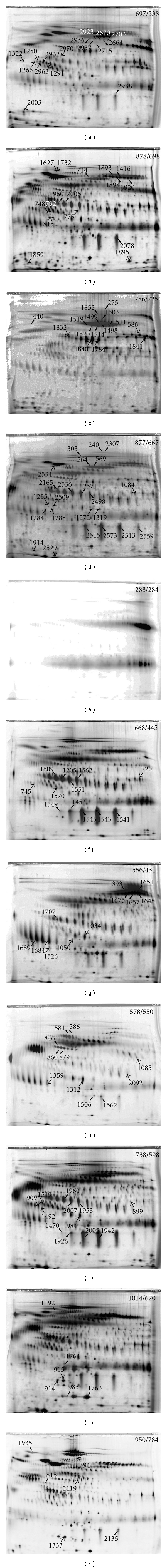
Localization of protein spots showing different intensities between the ProteoMinor-treated and untreated samples. Panels (a–k) correspond to those in Figure 3. The protein spot numbers corresponds to those in Supplementary Tables  5 and 6. −/−: number of protein spots without ProteoMiner treatment/those with ProteoMiner treatment.

The fluorescent 2D-PAGE images of the ProteoMiner-treated and untreated samples were overlaid with different colors, so that the unique protein contents were visualized (Figures 3 and 4). The results of experiments in which the fluorescent dyes were swapped are shown in Supplementary Figure 2. Consistent with the SDS-PAGE results (Figure 2), Figure 3 demonstrates that the approach involving depletion of high-abundance proteins and multidimensional separation was an effective prefractionation method to increase the number of protein spots, and the use of ProteoMiner treatment also contributed to reveal more plasma proteins. Because these fractionation methods are based on different binding properties of proteins, their combined use revealed additional plasma proteins.

The number of observed protein spots on 2D gel electrophoresis is summarized in Supplementary Table  2. Overall, the total number of protein spots increased by treating the samples with ProteoMiner, except for the bound fraction from the Protein G-Sepharose column. This observation suggests that ProteoMiner may be a useful tool to observe a greater number of protein spots in prefractionated samples. 

We compared the protein spots of the samples with and without ProteoMiner treatment (Supplementary Table  3). Depending on the criteria, different numbers of protein spots showed significantly different intensities. Although the total number of protein spots increased by treating the samples with ProteoMiner (Supplementary Table  2), many protein spots revealed decreased intensity with treatment, suggesting the selective enrichment by ProteoMiner.

### 3.5. Mass Spectrometric Identification of Proteins with Different Affinities to ProteoMiner

To reveal the characteristics of proteins with a particularly high or low affinity to ProteoMiner, among the protein spots with greater than 5-fold differences (Supplementary Table  3), we selected those with the top 10% different intensities between the ProteoMiner-treated and untreated samples in each fraction and subjected them to mass spectrometric identification. A total of 200 protein spots were subjected to mass spectrometry, and a positive identification was obtained for 128 (Supplementary Table  4). A list of the identified proteins is provided in Supplementary Table  5, and data supporting protein identification are shown in Supplementary Table  6. These 128 protein spots corresponded to 29 unique proteins. Because the fold difference of the protein spots in the bound fraction from the Protein G-Sepharose column was less than 4, we did not examine them. Of the original plasma samples, vitronectin and albumin were most affected by ProteoMiner treatment and disappeared after depletion and fractionation using the anion-exchange column. The other proteins were identified as enriched (or nonenriched) by ProteoMiner treatment. Proteins bound to ProteoMiner have been reported in previous studies in which the proteins were globally identified by mass spectrometry. Dwivedi et al. demonstrated that albumin, alpha 1-antitrypsin, alpha 2-macroglobulin, apolipoprotein A-I, apolipoprotein A-II, haptoglobin-related protein, and serotransferrin have high affinity to ProteoMiner [21]. In addition, Beseme et al. identified apolipoprotein A-IV, apolipoprotein D, apolipoprotein E, ceruloplasmin, complement C3, fibrinogen beta, fibrinogen gamma, ficolin-2, ficolin-3, paroxonase I, prothrombin, transthyretin, and vitronectin [23]. The protein concentrations identified in this study are summarized in Supplementary Table  6. According to the literatures, the identified proteins were classified as high- and medium-abundance proteins. Adiponectin and the carboxypeptidase N catalytic chain are not reported in previous studies, in which ProteoMiner-treated samples were examined by 2D-PAGE and mass spectrometry.

Adiponectin is an adipocytokine [24–27] and plays a protective role against obesity-related disorders such as metabolic syndrome [28], type 2 diabetes [29], and cardiovascular disease [30]. Low levels of plasma adiponectin are associated with obesity [31] and many types of malignancies such as liver cancer [32], breast cancer [33], pancreatic cancer [34], and endometrial cancer [35]. An epidemiological study suggested that adiponectin is involved in early colorectal carcinogenesis [36], and that a low circulating adiponectin level is correlated with a poor prognosis in patients with colorectal cancer [37]. The molecular backgrounds of these observations may be attributable to the antiproliferative effects of adiponectin on cancer cells [38]. 

Carboxypeptidase N (CPN), which is also known as kininase I, arginine carboxypeptidase, and anaphylatoxin inactivator, is a zinc finger metalloprotease. It cleaves basic lysine and arginine residues from the carboxy terminal of proteins [39]. CPN is produced in the liver and secreted into the plasma. It modulates the activity of cytokines such as stromal cell-derived factor-1 alpha [40]. The association of CPN1 with malignancy and other diseases has not been reported, and the clinical utility of CPN1 has not been suggested.

The working hypothesis of this study was that the combined use of different separation methods, including a solid-phase hexapeptide ligand library, would increase the number of observable proteins, and finally visualize the proteome that may not be observed otherwise. By loading a high amount of protein and using extensive prefractionation techniques prior to using ProteoMiner, trace proteins became visible in SDS-PAGE, and the number of protein spots on 2D-DIGE increased significantly. This approach may pave a way to a novel strategy for intact plasma proteomics. In contrast, the present results of mass spectrometric protein identification did not support the use of a solid-phase hexapeptide ligand library to enrich low-abundance proteins. It may be because our present approach had 3 limitations. First, mass spectrometric identification was performed for proteins with a greater prominent difference between the samples with or without ProteoMiner treatment, and only 128 of 200 proteins were successfully identified (Supplementary Table  4), probably because of the low protein amount. Proteins with a smaller difference or amount may include trace proteins. Although we optimized the protocols for mass spectrometric protein identification because the sensitivity of the fluorescent dye in the 2D-DIGE was very high, not all protein spots on 2D-DIGE could be identified by mass spectrometry. To evaluate enriched proteins, the complementary use of an LC-MS/MS shotgun approach may be worth considering. Second, proteins from ProteoMiner were recovered by a single-step elution with 8 M urea, 2% CHAPS, and 5% acetic acid, according to the manufacturer's instructions (Bio-Rad). However, because proteins may have interacted with hexapeptide ligand libraries in all possible modes, the absolute elution process may require sequential steps or more stringent buffer conditions such as boiling 10% SDS with 3% DTE [41]. Furthermore, various binding conditions may also be worth considering to capture whole binding proteins [9]. Third, considering the practical use of trace proteins in a biomarker study, we used as much sample as possible for identifying them and examined 10 mL plasma samples as an initial source. However, a larger volume of plasma sample, such as 100 mL, might be needed to collect rare proteins. In practice, such a high volume of plasma is rarely obtained for many cases in biomarker studies, and we may need to optimize the protocols for use of 10 mL plasma. For instance, we identify the biomarker candidates using 100 mL plasma, and using specific antibody against the identified candidate, we will be able to screen a relatively large number of samples with 10 mL volume or less.

The combined use of the ProteoMiner and the proteomic modalities in this study may enable the quantitative comparison for biomarker studies. We demonstrated that the liquid chromatography was quantitatively reproducible (Supplementary Figure 1), and the quantitative reproducibility of the ProteoMiner and 2D-DIGE was previously reported [10, 17]. We may further need to examine how the combined use of such reproducible methods generate the results in a reproducible way, considering the degree of differences that we expect between the samples to be compared.

## 4. Conclusions

The use of ProteoMiner in combination with conventional proteomic modalities such as depletion and anion-exchange columns significantly enhanced trace proteins on SDS-PAGE and increased the number of protein spots on 2D-DIGE, suggesting that the use of a solid-phase hexapeptide ligand library has great potential for intact plasma proteomics. Mass spectrometric protein identification revealed that high- and middle-abundance proteins were enriched by ProteoMiner, and the characteristics of proteins with unique affinity to a solid-phase hexapeptide ligand library remain to be clarified by more extensive mass spectrometric protein identification. Although use of ProteoMiner for biomarker studies is quite feasible and attractive, more extensive characterization of binding proteins and optimized protocols are required for large-scale biomarker studies.

## Supplementary Material

Supplementary table 1: Recovery rate of protein samples after ProteoMiner treatment.
Supplementary table 2: Number of protein spots from fractionated plasma samples.
Supplementary table 3: Number of protein spots between the ProteoMiner-treated and untreated protein samples with different criteria.
Supplementary table 4: Number of protein spots observed by mass spectrometric protein identification.
Supplementary table 5: List of proteins identified by mass spectrometry.
Supplementary table 6: Detailed data of identified proteins.
Supplementary table 7: List of the identified proteins and their reported concentrations and references.
Supplementary figure 1: Reproducibility of protein fractionation by liquid chromatography. The ultraviolet detection (280 nm) trace for each run demonstrated consistent separation and fractionation. A. HiTrap Blue HP column; B. HiTrap Protein G HP column; C. Resource Q column.
Supplementary figure 2: Two-dimensional difference gel electrophoresis images of ProteoMiner-treated and untreated protein samples. The ProteoMiner-treated and untreated samples were labeled with Cy5 and Cy3, respectively. A. original plasma; B. flow-through fraction of HiTrap Blue HP column; C. binding fraction of HiTrap Blue HP column; D. flow-through fraction of HiTrap Protein G HP column; E. binding fraction of HiTrap Protein G HP column; 0 mM fraction (F), 100 mM fraction (G), 150 mM (H), 200 mM (I), 250 mM (J), and 1 M fraction (K) of Resource Q column.

## Figures and Tables

**Table 1:** List of the identified proteins and their reported concentration.

Protein name	Normal concentration *μ*g/mL
Adiponectin	2–17
Albumin	35000–52000
Alpha-1-antitrypsin	900–2000
Alpha-1B-glycoprotein	150–300
Alpha-2-macroglobulin	1300–3000
Apolipoprotein A-I	1000–2000
Apolipoprotein A-II	190–300
Apolipoprotein A-IV	110–220
Apolipoprotein D	60–90
Apolipoprotein E	30–60
Carboxypeptidase N	30
Ceruloplasmin	190–370
Clusterin	250–420
Coagulation factor X	10
Complement C3	900–1800
Complement C4-A	25–90
Fibrinogen beta chain	520–1420
Fibrinogen gamma chain	490–1340
Ficolin-2	1–12
Ficolin-3	3–54
Haptoglobin	200–2000
Haptoglobin-related protein	32–41
Inter-alpha-trypsin inhibitor (heavy chain H3)	100–200
Paraoxonase/arylesterase 1	58–61
Prothrombin	100
Serotransferrin	2000–3600
Transthyretin	200–400
Vitronectin	240–530
Zinc-alpha-2-glycoprotein	60–80

The table with the references for the protein concentration is shown in Supplementary Table  7 in Supplemrntary material available online at doi: 10.1155/2011/39615.
