# Supplementary material for: Combined Use of a Solid-Phase Hexapeptide Ligand Library with Liquid Chromatography and Two-Dimensional Difference Gel Electrophoresis for Intact Plasma Proteomics
Source: Int J Proteomics. 2011 Sep 8;2011:739615. doi: 10.1155/2011/739615 (PMC3282153; doi:10.1155/2011/739615)

Legends for supplementary tables and figures

Supplementary table 1

Recovery rate of protein samples after ProteoMiner treatment

Supplementary table 2

Number of protein spots from fractionated plasma samples

Supplementary table 3

Number of protein spots between the ProteoMiner-treated and untreated protein samples with different criteria

Supplementary table 4

Number of protein spots observed by mass spectrometric protein identification

Supplementary table 5

List of proteins identified by mass spectrometry

Supplementary table 6

Detailed data of identified proteins

Supplementary table 7

List of the identified proteins and their reported concentrations and references

Supplementary figure 1

Reproducibility of protein fractionation by liquid chromatography. The ultraviolet detection (280 nm) trace for each run demonstrated consistent separation and fractionation. A. HiTrap Blue HP column ; B. HiTrap Protein G HP column ; C. Resource Q column.

Supplementary figure 2

Two-dimensional difference gel electrophoresis images of ProteoMiner-treated and untreated protein samples. The ProteoMiner-treated and untreated samples were labeled with Cy5 and Cy3, respectively. A. original plasma; B. flow-through fraction of HiTrap Blue HP column; C. binding fraction of HiTrap Blue HP column; D. flow-through fraction of HiTrap Protein G HP column; E. binding fraction of HiTrap Protein G HP column; 0 mM fraction (F), 100 mM fraction (G), 150 mM (H), 200 mM (I), 250 mM (J), and 1 M fraction (K) of Resource Q column.

Supplementary table 1 Recovery rate of protein samples after ProteoMiner treatment

fractions	samples subjected to ProteoMiner		samples purified by ProteoMiner		recovery rate (%)
	concentration (μg/μl)	amount (mg)	concentration (μg/μl)	amount (mg)	
plasma	61.80	61.80	6.08	1.22	1.97
Blue FT	169.66	169.66	7.27	1.45	0.86
Blue BD	250.60	250.60	16.77	3.35	1.34
ProG FT	57.96	57.96	8.31	1.66	2.87
ProG BD	96.12	19.22	10.57	0.42	2.20
0 mM NaCl elute	56.54	11.31	4.52	0.18	1.60
100 mM NaCl elute	83.48	16.70	3.92	0.16	0.94
150 mM NaCl elute	109.70	21.94	3.15	0.13	0.57
200 mM NaCl elute	116.90	23.38	3.73	0.15	0.64
250 mM NaCl elute	20.50	4.10	4.91	0.20	4.80
1 M NaCl elute	19.60	3.92	6.20	0.25	6.33

Supplementary table 2 Number of protein spots from fractionated plasma samples

fractions	number of protein spots with ProteoMiner treatment	number of protein spots without ProteoMiner treatment
plasma	697	538
Blue FT	878	698
Blue BD	786	725
Protein G FT	877	667
Protein G BD	288	284
0 mM NaCl elute	668	445
100 mM NaCl elute	556	431
150 mM NaCl elute	578	550
200 mM NaCl elute	738	598
250 mM NaCl elute	1014	670
1 M NaCl elute	950	784
total	8030	6390

Table 3 Number of protein spots between the ProteoMiner-treated and untreated protein samples with different criteria

fractions	>2-fold[1)]		>4-fold[1)]		>5-fold[1)]		>8-fold[1)]		>10-fold[1)]		>20-fold[1)]	
	increase[2)]	decrease[2)]	increase	decrease	increase	decrease	increase	decrease	increase	decrease	increase	decrease
plasma	259	246	183	119	153	83	67	54	36	46	9	18
Blue FT	387	266	245	174	207	128	120	73	97	57	25	21
Blue BD	241	215	120	146	91	118	47	76	35	66	5	36
ProG FT	352	250	228	169	176	149	98	99	58	79	8	44
ProG BD	40	17	1	0	0	0	0	0	0	0	0	0
0 mM NaCl elute	233	123	101	104	61	95	31	68	9	51	0	16
100 mM NaCl elute	211	130	114	79	95	65	60	53	45	43	6	18
150 mM NaCl elute	211	192	100	104	69	82	35	47	23	37	6	9
200 mM NaCl elute	239	192	102	100	74	84	36	47	16	32	1	15
250 mM NaCl elute	311	241	109	108	76	73	27	31	22	19	7	2
1 M NaCl elute	216	184	83	74	67	37	40	3	24	2	8	2
subtotal	2700	2056	1386	1177	1069	914	561	551	365	432	75	181
total	4756		2563		1983		1112		797		256	

[1)]the ratio of intensity of protein spots (ProteoMiner treated / ProteoMiner not-treated).

[2)]increase and decrease: the protein spots with the increased and decreased intensity after ProteoMiner treatment, respectively.

Supplementary table 4 Number of protein spots observed by mass spectrometric protein identification

fractions	A[1)]	B[2)]
plasma	8/15[3)]	8/8
Blue FT	8/21	8/13
Blue BD	5/9	10/12
ProG FT	10/18	13/15
ProG BD	-/-	-/-
0 mM	3/6	9/10
100 mM	5/10	6/7
150 mM	4/7	7/8
200 mM	5/7	7/8
250 mM	2/8	4/7
1 M	3/7	3/4
subtotal	53/108	75/92
total	128/200	

[1)]A: Protein spots with increased intensity by ProteoMiner treatment.

[2)]B: Protein spots with decreased intensity by ProteoMiner treatment.

[3)] Protein spots with positive identification by massspectrometry / protein spots examined by mass spectrometry.

Supplementary table 5 List of proteins identified by mass spectrometry

plasma

spot number [1]	accession number [2]	protein name [2]	number of matched peptides	sequence coverage (%) [3]	MASCOT score [4]	fold difference [5]
1250	P04004	vitronectin	3	9.0	182	16.5
1266	P04004	vitronectin	2	6.5	127	20.1
1291	P04004	vitronectin	3	9.0	220	21.4
1322	P04004	vitronectin	2	5.6	117	17.7
2003	P0C0L4	complement C4-A	2	1.2	118	71.0
2664	P02768	albumin	28	41.4	1546	0.026
2670	P02768	albumin	24	37.8	1310	0.024
2703	P02768	albumin	28	40.2	1521	0.024
2715	P02768	albumin	22	36.8	1096	0.029
2923	P02768	albumin	24	38.6	1278	0.020
2936	P02768	albumin	20	33.7	1045	0.021
2937	P02768	albumin	24	38.6	1267	0.019
2938	P02768	albumin	5	10.8	259	0.023
2962	P04004	vitronectin	3	9.0	195	47.5
2963	P04004	vitronectin	3	9.0	172	52.9
2970	P01024	complement C3	13	10.4	627	20.0

Blue FT

spot number	accession number	protein name	number of matched peptides	sequence coverage (%)	MASCOT score	fold difference
775	P06727	apolipoprotein A-IV	5	15.7	237	36.4
792	P10909	clusterin	2	8.9	118	27.9
813	P27169	paraoxonase/arylesterase 1	6	24.5	347	25.5
1416	P02787	serotransferrin	11	21.2	696	0.034
1627	P00734	prothrombin	6	11.7	367	39.2
1732	P00734	prothrombin	6	11.7	326	24.3
1744	P02768	albumin	16	23.8	621	0.031
1748	P01009	alpha-1-antitrypsin	5	19.9	229	44.5
1859	P02647	apolipoprotein A-I	5	20.2	290	39.2
1893	P02787	serotransferrin	9	14.6	534	0.026
1894	P02787	serotransferrin	13	22.6	850	0.027
1895	P0C0L4	complement C4-A	2	2.0	97	44.5
1920	P02787	serotransferrin	11	16.2	661	0.021
1960	P00738	haptoglobin	12	20.9	606	0.017
2006	P00738	haptoglobin	8	16.5	426	0.028
2078	P00739	haptoglobin-related protein	2	6.3	124	0.015

Blue BD

spot number	accession	protein name	number of	sequence	MASCOT	fold

	number		matched peptides	coverage (%)	score	difference
275	P02768	albumin	8	13.8	413	0.015
440	P02768	albumin	4	9.0	153	0.016
586	P02675	fibrinogen beta chain	16	43.2	1009	24.8
1498	P02768	albumin	18	24.6	960	0.004
1499	P02768	albumin	25	38.8	1354	0.007
1503	P02768	albumin	19	30.4	772	0.014
1511	P02768	albumin	22	28.4	1142	0.005
1514	P02768	albumin	21	25.8	1112	0.004
1519	P02768	albumin	26	29.4	1233	0.003
1520	P02768	albumin	7	12.3	430	0.007
1784	P02679	fibrinogen gamma chain	19	47.7	1164	22.3
1832	P02679	fibrinogen gamma chain	19	47.9	1078	21.4
1840	P02679	fibrinogen gamma chain	19	45.5	1109	19.0
1841	P02675	fibrinogen beta chain	15	40.7	952	19.1
1852	P02768	albumin	10	16.6	590	0.016

ProG FT

spot number	accession number	protein name	number of matched peptides	sequence coverage (%)	MASCOT score	fold difference
240	P01023	alpha-2-macroglobulin	31	28.1	1890	0.029
303	P00450	ceruloplasmin	27	36.2	1538	0.027
564	P02768	albumin	12	18.4	647	0.027
569	P02768	albumin	13	18.1	721	0.026
1084	Q15485	ficolin-2	3	10.2	195	24.4
1255	P27169	paraoxonase/arylesterase 1	5	18.3	283	45.3
1272	P00734	prothrombin	2	3.5	131	16.5
1284	P27169	paraoxonase/arylesterase 1	5	18.3	270	18.5
1285	P27169	paraoxonase/arylesterase 1	5	18.3	280	21.9
1319	P02649	apolipoprotein E	7	28.7	485	16.5
1914	P02647	apolipoprotein A-I	7	25.5	415	17.6
2165	P00738	haptoglobin	10	19.5	522	0.031
2307	P01023	alpha-2-macroglobulin	24	21.4	1488	0.032
2498	P00738	haptoglobin	9	16.0	447	0.027
2509	P27169	paraoxonase/arylesterase 1	5	18.3	309	20.4
2513	P00738	haptoglobin	4	6.9	241	0.009
2515	P00739	haptoglobin-related protein	2	3.7	82	0.022
2529	P02652	apolipoprotein A-II	3	21.0	140	25.6
2534	P00734	prothrombin	6	11.7	380	17.8
2536	P06727	apolipoprotein A-IV	12	33.1	661	0.019
2559	P00738	haptoglobin	5	10.8	306	0.025
2571	P00738	haptoglobin	10	17.5	513	0.022
2573	P00738	haptoglobin	10	15.3	543	0.021

0 mM NaCl elute

spot number	accession number	protein name	number of matched peptides	sequence coverage (%)	MASCOT score	fold difference
720	P00738	haptoglobin	5	12.1	279	12.2
745	P10909	clusterin	8	18.0	354	10.8
1205	P00738	haptoglobin	13	24.4	662	0.022
1452	P00738	haptoglobin	10	18.7	593	0.018
1509	P00738	haptoglobin	10	22.7	543	0.021
1541	P00738	haptoglobin	11	19.0	605	0.025
1543	P00738	haptoglobin	8	12.3	445	0.023
1545	P00738	haptoglobin	6	8.1	326	0.019
1549	P00739	haptoglobin-related protein	2	3.7	152	0.020
1551	P00738	haptoglobin	13	26.6	722	0.023
1562	P00738	haptoglobin	15	31.3	785	0.027
1570	P10909	clusterin	4	10.9	174	10.8

100 mM NaCl elute

spot number	accession number	protein name	number of matched peptides	sequence coverage (%)	MASCOT score	fold difference
1034	Q15848	adiponectin	3	21.7	167	20.6
1050	P01009	alpha-1-antitrypsin	8	19.6	402	22.6
1393	P02787	serotransferrin	14	21.9	874	0.027
1526	P05090	apolipoprotein D	2	12.7	87	19.8
1648	P02787	serotransferrin	16	22.3	922	0.025
1651	P02787	serotransferrin	18	28.4	1218	0.019
1657	P02787	serotransferrin	17	25.8	1064	0.022
1675	P02787	serotransferrin	18	28.4	1055	0.023
1684	P05090	apolipoprotein D	2	12.7	78	24.8
1689	P05090	apolipoprotein D	2	12.7	86	20.6
1707	P25311	zinc-alpha-2-glycoprotein	14	46.1	713	0.026

150 mM NaCl elute

spot number	accession number	protein name	number of matched peptides	sequence coverage (%)	MASCOT score	fold difference
581	P04217	alpha-1B-glycoprotein	16	29.5	774	0.038
586	P04217	alpha-1B-glycoprotein	16	23.8	565	0.031
846	P01009	alpha-1-antitrypsin	87	56.9	1587	0.043
860	P01009	alpha-1-antitrypsin	114	59.1	1709	0.032
879	P01009	alpha-1-antitrypsin	17	42.8	778	0.035
1085	P15169	carboxypeptidase N catalytic chain	4	13.1	250	22.9
1312	Q15848	adiponectin	5	29.5	222	22.6
1359	P05090	apolipoprotein D	2	12.7	92	18.7
1506	P00739	haptoglobin-related protein	6	4.3	194	0.032

1562	P00738	haptoglobin	8	6.9	201	0.042
2092	O75636	ficolin-3	5	17.7	242	22.7

200 mM NaCl elute

spot number	accession number	protein name	number of matched peptides	sequence coverage (%)	MASCOT score	fold difference
899	Q15485	ficolin-2	3	14.7	193	13.2
909	P10909	clusterin	9	18.7	465	13.2
984	Q15848	adiponectin	4	29.5	198	20.9
1470	P02766	transthyretin	7	63.3	375	0.038
1492	P10909	clusterin	8	18.5	369	13.6
1519	P10909	clusterin	9	21.8	461	15.4
1926	P02647	apolipoprotein A-I	7	32.2	465	0.020
1942	P02647	apolipoprotein A-I	7	32.2	456	0.035
1953	P02766	transthyretin	5	54.4	237	0.039
1960	P02766	transthyretin	16	65.3	568	0.035
2005	P02647	apolipoprotein A-I	3	13.5	241	0.033
2007	P02766	transthyretin	8	50.3	236	0.039

250 mM NaCl elute

spot number	accession number	protein name	number of matched peptides	sequence coverage (%)	MASCOT score	fold difference
914	P00450	ceruloplasmin	7	4.1	216	53.5
915	P00450	ceruloplasmin	7	4.1	226	32.5
983	P02768	albumin	25	24.6	1066	0.068
1192	P00738	haptoglobin	6	6.9	199	0.033
1763	P06727	apolipoprotein A-IV	16	39.1	783	0.065
1764	P00738	haptoglobin	10	18.2	441	0.055

1 M NaCl elute

spot number	accession number	protein name	number of matched peptides	sequence coverage (%)	MASCOT score	fold difference
394	P00450	ceruloplasmin	41	34.8	1648	21.1
815	P00742	coagulation factor X	8	10.2	238	0.116
1333	P00450	ceruloplasmin	6	5.5	218	212.1
1935	Q06033	inter-alpha-trypsin inhibitor heavy chain H3	38	26.1	1135	0.042
2119	P00450	ceruloplasmin	15	13.1	599	26.2
2135	P00738	haptoglobin	2	6.2	105	0.128

[1)]Spot numbers refer to those in Figure 5.

[2)]Accession numbers of proteins and protein name were derived from Swiss-Prot and NCBI nonredundant datebases.

[3)]Percentage of the number of the identified amino acids per those of total ones.

[4)]MASCOT score for the identified proteins based on the peptide ions score (P<0.05) (http://www.matrixscience.com).

[5)]The ratio of intensity of protein spots between ProteoMiner treated and ProteoMiner not-treated samples.

Supplementary table 6 Detailed data of identified proteins

Spots No.[1]	Rank	Accession No.[2]	protein ID[2]	Identified protein[2]	pI (cal)[3]	MW (cal) (D)[3]	Protein score[4]	Peptide matches	Sequence coverage (%)[5]	Ion charge state (+)	MZ (obs)[6]	Mass (obs)[7]	Mass (calc)[8]	Delta[9]	Miss[10]	Mascot ions score[11]	Peptide sequence	Modification
1250	1	P04004	VTNC	Vitronectin	5.55	55069	182	3	9.0	2	712.1611	1422.3076	1421.6463	0.6613	0	77.66	FEDGVLDPDYPR	
	1	P04004	VTNC	Vitronectin	5.55	55069	182	3	9.0	2	824.4891	1646.9636	1645.81	1.1536	0	54.82	DVWGIEGPIDAAFTR	
	1	P04004	VTNC	Vitronectin	5.55	55069	182	3	9.0	3	556.5422	1666.6048	1665.7682	0.8366	0	52.49	DWHGVPGQVDAAMAGR	
1266	1	P04004	VTNC	Vitronectin	5.55	55069	127	2	6.5	2	824.2292	1646.4438	1645.81	0.6338	0	74	DVWGIEGPIDAAFTR	
	1	P04004	VTNC	Vitronectin	5.55	55069	127	2	6.5	3	556.4452	1666.3138	1665.7682	0.5456	0	56	DWHGVPGQVDAAMAGR	
1291	1	P04004	VTNC	Vitronectin	5.55	55069	220	7	9.0	2	711.9957	1421.9768	1421.6463	0.3305	0	39	FEDGVLDPDYPR	
	1	P04004	VTNC	Vitronectin	5.55	55069	220	7	9.0	2	712.2281	1422.4416	1421.6463	0.7953	0	91	FEDGVLDPDYPR	
	1	P04004	VTNC	Vitronectin	5.55	55069	220	7	9.0	2	824.2217	1646.4288	1645.81	0.6188	0	64	DVWGIEGPIDAAFTR	
	1	P04004	VTNC	Vitronectin	5.55	55069	220	7	9.0	2	824.3691	1646.7236	1645.81	0.9136	0	61	DVWGIEGPIDAAFTR	
	1	P04004	VTNC	Vitronectin	5.55	55069	220	7	9.0	3	556.5002	1666.4788	1665.7682	0.7106	0	56	DWHGVPGQVDAAMAGR	
	1	P04004	VTNC	Vitronectin	5.55	55069	220	7	9.0	3	561.8622	1682.5648	1681.7631	0.8017	0	68	DWHGVPGQVDAAMAGR	Oxidation (M)

	1	P04004	VTNC	Vitronectin	5.55	55069	220	7	9.0	3	561.8812	1682.6218	1681.7631	0.8587	0	42	DWHGVPGQVDAAMAGR	Oxidation (M)
	2	P10909	CLUS	Clusterin	5.89	53031	153	3	6.9	2	538.1241	1074.2336	1073.5353	0.6984	0	38	IDSLLENDR	
	2	P10909	CLUS	Clusterin	5.89	53031	153	3	6.9	2	559.4366	1116.8586	1116.6026	0.256	0	46	TLLSNLEEAK	
	2	P10909	CLUS	Clusterin	5.89	53031	153	3	6.9	2	697.5226	1393.0306	1392.6885	0.3422	0	75	ASSIIDELFQDR	
1322	1	P04004	VTNC	Vitronectin	5.55	55069	117	2	5.6	2	712.1891	1422.3636	1421.6463	0.7173	0	58	FEDGVLDPDYPR	
	1	P04004	VTNC	Vitronectin	5.55	55069	117	2	5.6	2	824.4347	1646.8548	1645.81	1.0448	0	62	DVWGIEGPIDAAFTR	
2003	1	P0C0L4	CO4A	Complement C4-A	6.65	194247	118	4	1.2	1	983.554	982.5467	982.4794	0.0674	0	34	TYNVLDMK	
	1	P0C0L4	CO4A	Complement C4-A	6.65	194247	118	4	1.2	2	492.3926	982.7706	982.4794	0.2913	0	50	TYNVLDMK	
	1	P0C0L4	CO4A	Complement C4-A	6.65	194247	118	4	1.2	2	500.5446	999.0746	998.4743	0.6004	0	37	TYNVLDMK	Oxidation (M)
	1	P0C0L4	CO4A	Complement C4-A	6.65	194247	118	4	1.2	2	719.2596	1436.5046	1435.7195	0.7852	0	71	GLEEELQFSLGSK	
	1	P0C0L5	CO4B	Complement C4-B	6.73	194212	118	4	1.2	1	983.554	982.5467	982.4794	0.0674	0	34	TYNVLDMK	
	1	P0C0L5	CO4B	Complement C4-B	6.73	194212	118	4	1.2	2	492.3926	982.7706	982.4794	0.2913	0	50	TYNVLDMK	
	1	P0C0L5	CO4B	Complement C4-B	6.73	194212	118	4	1.2	2	500.5446	999.0746	998.4743	0.6004	0	37	TYNVLDMK	Oxidation (M)
	1	P0C0L5	CO4B	Complement C4-B	6.73	194212	118	4	1.2	2	719.2596	1436.5046	1435.7195	0.7852	0	71	GLEEELQFSLGSK	

2664	1	P02768	ALBU	Albumin	5.92	71317	1546	101	41.4	1	695.487	694.4797	694.3286	0.1512	0	35	NYAEAK
	1	P02768	ALBU	Albumin	5.92	71317	1546	101	41.4	1	880.546	879.5387	879.4338	0.1049	0	43	AEFAEVSK
	1	P02768	ALBU	Albumin	5.92	71317	1546	101	41.4	2	464.5271	927.0396	926.4861	0.5535	0	36	YLYEIAR
	1	P02768	ALBU	Albumin	5.92	71317	1546	101	41.4	1	940.585	939.5777	939.441	0.1367	0	47	DDNPNLPR
	1	P02768	ALBU	Albumin	5.92	71317	1546	101	41.4	2	471.0721	940.1296	939.441	0.6887	0	36	DDNPNLPR
	1	P02768	ALBU	Albumin	5.92	71317	1546	101	41.4	1	951.404	950.3967	950.4345	-0.0378	0	45	DLGEENFK
	1	P02768	ALBU	Albumin	5.92	71317	1546	101	41.4	2	480.8531	959.6916	959.5552	0.1364	0	47	FQNALLVR
	1	P02768	ALBU	Albumin	5.92	71317	1546	101	41.4	2	480.8801	959.7456	959.5552	0.1904	0	49	FQNALLVR
	1	P02768	ALBU	Albumin	5.92	71317	1546	101	41.4	2	481.1921	960.3696	959.5552	0.8144	0	62	FQNALLVR
	1	P02768	ALBU	Albumin	5.92	71317	1546	101	41.4	2	481.5561	961.0976	959.5552	1.5424	0	46	FQNALLVR
	1	P02768	ALBU	Albumin	5.92	71317	1546	101	41.4	2	492.8071	983.5996	983.4811	0.1185	0	36	TYETTLEK
	1	P02768	ALBU	Albumin	5.92	71317	1546	101	41.4	2	493.0811	984.1476	983.4811	0.6665	0	34	TYETTLEK
	1	P02768	ALBU	Albumin	5.92	71317	1546	101	41.4	2	500.9726	999.9306	999.5964	0.3342	0	59	QTALVELVK
	1	P02768	ALBU	Albumin	5.92	71317	1546	101	41.4	2	501.1006	1000.1866	999.5964	0.5902	0	61	QTALVELVK

1	P02768	ALBU	Albumin	5.92	71317	1546	101	41.4	2	507.5711	1013.1276	1012.5917	0.536	0	86	LVAASQAALGL
1	P02768	ALBU	Albumin	5.92	71317	1546	101	41.4	2	507.6491	1013.2836	1012.5917	0.692	0	95	LVAASQAALGL
1	P02768	ALBU	Albumin	5.92	71317	1546	101	41.4	2	507.8966	1013.7786	1012.5917	1.187	0	44	LVAASQAALGL
1	P02768	ALBU	Albumin	5.92	71317	1546	101	41.4	1	1017.59	1016.5827	1016.5291	0.0536	0	42	SLHTLFGDK
1	P02768	ALBU	Albumin	5.92	71317	1546	101	41.4	2	509.4081	1016.8016	1016.5291	0.2725	0	45	SLHTLFGDK
1	P02768	ALBU	Albumin	5.92	71317	1546	101	41.4	2	528.7577	1055.5008	1054.5811	0.9198	1	54	KYLYEIAR
1	P02768	ALBU	Albumin	5.92	71317	1546	101	41.4	2	537.8602	1073.7058	1073.5353	0.1706	1	44	LDELRDEGK
1	P02768	ALBU	Albumin	5.92	71317	1546	101	41.4	1	1128.705	1127.6977	1127.6914	0.0063	1	36	KQTALVELVK
1	P02768	ALBU	Albumin	5.92	71317	1546	101	41.4	2	565.1052	1128.1958	1127.6914	0.5045	1	67	KQTALVELVK
1	P02768	ALBU	Albumin	5.92	71317	1546	101	41.4	2	565.1531	1128.2916	1127.6914	0.6003	1	52	KQTALVELVK
1	P02768	ALBU	Albumin	5.92	71317	1546	101	41.4	2	571.6417	1141.2688	1140.6866	0.5822	1	78	KLVAASQAALGL
1	P02768	ALBU	Albumin	5.92	71317	1546	101	41.4	2	575.6157	1149.2168	1148.6077	0.6091	0	54	LVNEVTEFAK
1	P02768	ALBU	Albumin	5.92	71317	1546	101	41.4	2	575.6232	1149.2318	1148.6077	0.6241	0	55	LVNEVTEFAK
1	P02768	ALBU	Albumin	5.92	71317	1546	101	41.4	2	576.0906	1150.1666	1148.6077	1.5589	0	51	LVNEVTEFAK

1	P02768	ALBU	Albumin	5.92	71317	1546	101	41.4	2	576.2886	1150.5626	1148.6077	1.9549	0	37	LVNEVTEFAK	
1	P02768	ALBU	Albumin	5.92	71317	1546	101	41.4	2	613.9222	1225.8298	1225.5979	0.232	1	44	FKDLGEENFK	
1	P02768	ALBU	Albumin	5.92	71317	1546	101	41.4	2	614.1317	1226.2488	1225.5979	0.651	1	41	FKDLGEENFK	
1	P02768	ALBU	Albumin	5.92	71317	1546	101	41.4	1	1311.823	1310.8157	1310.7347	0.0811	0	35	HPDYSVVLLLR	
1	P02768	ALBU	Albumin	5.92	71317	1546	101	41.4	2	656.8282	1311.6418	1310.7347	0.9072	0	51	HPDYSVVLLLR	
1	P02768	ALBU	Albumin	5.92	71317	1546	101	41.4	2	671.1722	1340.3298	1341.6275	-1.2976	0	58	AVMDDFAAFVEK	
1	P02768	ALBU	Albumin	5.92	71317	1546	101	41.4	2	671.8167	1341.6188	1341.6275	-0.0086	0	34	AVMDDFAAFVEK	
1	P02768	ALBU	Albumin	5.92	71317	1546	101	41.4	2	671.9911	1341.9676	1341.6275	0.3402	0	63	AVMDDFAAFVEK	
1	P02768	ALBU	Albumin	5.92	71317	1546	101	41.4	2	672.0632	1342.1118	1341.6275	0.4844	0	65	AVMDDFAAFVEK	
1	P02768	ALBU	Albumin	5.92	71317	1546	101	41.4	1	1343.619	1342.6117	1341.6275	0.9842	0	45	AVMDDFAAFVEK	
1	P02768	ALBU	Albumin	5.92	71317	1546	101	41.4	2	672.6877	1343.3608	1341.6275	1.7334	0	69	AVMDDFAAFVEK	
1	P02768	ALBU	Albumin	5.92	71317	1546	101	41.4	1	1358.592	1357.5847	1357.6224	-0.0377	0	46	AVMDDFAAFVEK	Oxidation (M)
1	P02768	ALBU	Albumin	5.92	71317	1546	101	41.4	2	680.1127	1358.2108	1357.6224	0.5884	0	53	AVMDDFAAFVEK	Oxidation (M)
1	P02768	ALBU	Albumin	5.92	71317	1546	101	41.4	2	680.1962	1358.3778	1357.6224	0.7554	0	61	AVMDDFAAFVEK	Oxidation (M)

1	P02768	ALBU	Albumin	5.92	71317	1546	101	41.4	2	734.5356	1467.0566	1466.8358	0.2209	1	45	RHPDYSVVLLLR
1	P02768	ALBU	Albumin	5.92	71317	1546	101	41.4	3	490.1355	1467.3847	1466.8358	0.5489	1	56	RHPDYSVVLLLR
1	P02768	ALBU	Albumin	5.92	71317	1546	101	41.4	3	490.1482	1467.4228	1466.8358	0.587	1	44	RHPDYSVVLLLR
1	P02768	ALBU	Albumin	5.92	71317	1546	101	41.4	3	490.1695	1467.4867	1466.8358	0.6509	1	45	RHPDYSVVLLLR
1	P02768	ALBU	Albumin	5.92	71317	1546	101	41.4	3	490.1892	1467.5458	1466.8358	0.71	1	55	RHPDYSVVLLLR
1	P02768	ALBU	Albumin	5.92	71317	1546	101	41.4	3	490.1978	1467.5716	1466.8358	0.7358	1	47	RHPDYSVVLLLR
1	P02768	ALBU	Albumin	5.92	71317	1546	101	41.4	3	490.2125	1467.6157	1466.8358	0.7799	1	52	RHPDYSVVLLLR
1	P02768	ALBU	Albumin	5.92	71317	1546	101	41.4	3	490.2515	1467.7327	1466.8358	0.8969	1	46	RHPDYSVVLLLR
1	P02768	ALBU	Albumin	5.92	71317	1546	101	41.4	3	490.2732	1467.7978	1466.8358	0.962	1	63	RHPDYSVVLLLR
1	P02768	ALBU	Albumin	5.92	71317	1546	101	41.4	3	490.2912	1467.8518	1466.8358	1.016	1	53	RHPDYSVVLLLR
1	P02768	ALBU	Albumin	5.92	71317	1546	101	41.4	3	490.3302	1467.9688	1466.8358	1.133	1	53	RHPDYSVVLLLR
1	P02768	ALBU	Albumin	5.92	71317	1546	101	41.4	3	504.9535	1511.8387	1510.8355	1.0031	0	43	VPQVSTPTLVEVSR
1	P02768	ALBU	Albumin	5.92	71317	1546	101	41.4	2	757.0912	1512.1678	1510.8355	1.3323	0	47	VPQVSTPTLVEVSR
1	P02768	ALBU	Albumin	5.92	71317	1546	101	41.4	2	757.2097	1512.4048	1510.8355	1.5693	0	71	VPQVSTPTLVEVSR

1	P02768	ALBU	Albumin	5.92	71317	1546	101	41.4	1	1623.787	1622.7797	1622.7803	-0.0006	0	51	DVFLGMFLYEYAR	
1	P02768	ALBU	Albumin	5.92	71317	1546	101	41.4	2	812.5327	1623.0508	1622.7803	0.2706	0	70	DVFLGMFLYEYAR	
1	P02768	ALBU	Albumin	5.92	71317	1546	101	41.4	2	812.5977	1623.1808	1622.7803	0.4006	0	70	DVFLGMFLYEYAR	
1	P02768	ALBU	Albumin	5.92	71317	1546	101	41.4	2	812.6262	1623.2378	1622.7803	0.4576	0	70	DVFLGMFLYEYAR	
1	P02768	ALBU	Albumin	5.92	71317	1546	101	41.4	2	812.6672	1623.3198	1622.7803	0.5396	0	70	DVFLGMFLYEYAR	
1	P02768	ALBU	Albumin	5.92	71317	1546	101	41.4	2	812.7147	1623.4148	1622.7803	0.6346	0	58	DVFLGMFLYEYAR	
1	P02768	ALBU	Albumin	5.92	71317	1546	101	41.4	2	812.7822	1623.5498	1622.7803	0.7696	0	70	DVFLGMFLYEYAR	
1	P02768	ALBU	Albumin	5.92	71317	1546	101	41.4	2	812.9382	1623.8618	1622.7803	1.0816	0	50	DVFLGMFLYEYAR	
1	P02768	ALBU	Albumin	5.92	71317	1546	101	41.4	2	813.0372	1624.0598	1622.7803	1.2796	0	69	DVFLGMFLYEYAR	
1	P02768	ALBU	Albumin	5.92	71317	1546	101	41.4	2	813.3347	1624.6548	1622.7803	1.8746	0	59	DVFLGMFLYEYAR	
1	P02768	ALBU	Albumin	5.92	71317	1546	101	41.4	2	820.5536	1639.0926	1638.7752	0.3174	0	40	DVFLGMFLYEYAR	Oxidation (M)
1	P02768	ALBU	Albumin	5.92	71317	1546	101	41.4	2	820.8746	1639.7346	1638.9305	0.8042	1	75	KVPQVSTPTLVEVSR	
1	P02768	ALBU	Albumin	5.92	71317	1546	101	41.4	2	820.9772	1639.9398	1638.9305	1.0094	1	78	KVPQVSTPTLVEVSR	
1	P02768	ALBU	Albumin	5.92	71317	1546	101	41.4	3	547.7579	1640.2519	1638.9305	1.3214	1	64	KVPQVSTPTLVEVSR	

1	P02768	ALBU	Albumin	5.92	71317	1546	101	41.4	3	547.8018	1640.3836	1638.9305	1.4531	1	43	KVPQVSTPTLVEVSR	
1	P02768	ALBU	Albumin	5.92	71317	1546	101	41.4	3	547.8385	1640.4937	1638.9305	1.5632	1	60	KVPQVSTPTLVEVSR	
1	P02768	ALBU	Albumin	5.92	71317	1546	101	41.4	2	821.2991	1640.5836	1638.7752	1.8084	0	69	DVFLGMFLYEYAR	Oxidation (M)
1	P02768	ALBU	Albumin	5.92	71317	1546	101	41.4	2	828.5397	1655.0648	1656.7453	-1.6805	0	41	QNCELFEQLGEYK	
1	P02768	ALBU	Albumin	5.92	71317	1546	101	41.4	3	581.4555	1741.3447	1741.8868	-0.5421	0	57	HPYFYAPELLFFAK	
1	P02768	ALBU	Albumin	5.92	71317	1546	101	41.4	2	872.4202	1742.8258	1741.8868	0.9391	0	48	HPYFYAPELLFFAK	
1	P02768	ALBU	Albumin	5.92	71317	1546	101	41.4	3	633.9352	1898.7838	1897.9879	0.7959	1	51	RHPYFYAPELLFFAK	
1	P02768	ALBU	Albumin	5.92	71317	1546	101	41.4	3	682.4075	2044.2007	2044.0881	0.1126	0	43	VFDEFKPLVEEPQNLIK	
1	P02768	ALBU	Albumin	5.92	71317	1546	101	41.4	3	682.6312	2044.8718	2044.0881	0.7837	0	37	VFDEFKPLVEEPQNLIK	
1	P02768	ALBU	Albumin	5.92	71317	1546	101	41.4	2	1023.46	2044.9054	2044.0881	0.8174	0	46	VFDEFKPLVEEPQNLIK	
1	P02768	ALBU	Albumin	5.92	71317	1546	101	41.4	3	682.6498	2044.9276	2044.0881	0.8395	0	50	VFDEFKPLVEEPQNLIK	
1	P02768	ALBU	Albumin	5.92	71317	1546	101	41.4	3	682.7148	2045.1226	2044.0881	1.0345	0	43	VFDEFKPLVEEPQNLIK	
1	P02768	ALBU	Albumin	5.92	71317	1546	101	41.4	3	682.7475	2045.2207	2044.0881	1.1326	0	43	VFDEFKPLVEEPQNLIK	
1	P02768	ALBU	Albumin	5.92	71317	1546	101	41.4	3	682.8125	2045.4157	2044.0881	1.3276	0	44	VFDEFKPLVEEPQNLIK	

1	P02768	ALBU	Albumin	5.92	71317	1546	101	41.4	3	682.8718	2045.5936	2044.0881	1.50555	0	35	VFDEFKPLVEEPQNLIK	
1	P02768	ALBU	Albumin	5.92	71317	1546	101	41.4	2	1023.885	2045.7554	2044.0881	1.6674	0	50	VFDEFKPLVEEPQNLIK	
1	P02768	ALBU	Albumin	5.92	71317	1546	101	41.4	2	1332.93	2663.8454	2665.2516	-1.4062	0	56	LVRPEVDVMCTAFHDNEETFLK	Oxidation (M)
1	P02768	ALBU	Albumin	5.92	71317	1546	101	41.4	3	889.0688	2664.1846	2665.2516	-1.0671	0	41	LVRPEVDVMCTAFHDNEETFLK	Oxidation (M)
1	P02768	ALBU	Albumin	5.92	71317	1546	101	41.4	3	889.4695	2665.3867	2665.2516	0.135	0	55	LVRPEVDVMCTAFHDNEETFLK	Oxidation (M)
1	P02768	ALBU	Albumin	5.92	71317	1546	101	41.4	3	890.0038	2666.9896	2665.2516	1.7379	0	39	LVRPEVDVMCTAFHDNEETFLK	Oxidation (M)
1	P02768	ALBU	Albumin	5.92	71317	1546	101	41.4	3	997.0201	2988.0385	2989.3321	-1.2936	0	59	SHCIAEVENDEMPADLPSLAADFVESK	Oxidation (M)
1	P02768	ALBU	Albumin	5.92	71317	1546	101	41.4	3	997.2101	2988.6085	2989.3321	-0.7236	0	63	SHCIAEVENDEMPADLPSLAADFVESK	Oxidation (M)
1	P02768	ALBU	Albumin	5.92	71317	1546	101	41.4	3	997.2352	2988.6838	2989.3321	-0.6483	0	52	SHCIAEVENDEMPADLPSLAADFVESK	Oxidation (M)
1	P02768	ALBU	Albumin	5.92	71317	1546	101	41.4	3	997.2889	2988.8449	2989.3321	-0.4872	0	39	SHCIAEVENDEMPADLPSLAADFVESK	Oxidation (M)
1	P02768	ALBU	Albumin	5.92	71317	1546	101	41.4	3	997.2995	2988.8767	2989.3321	-0.4554	0	35	SHCIAEVENDEMPADLPSLAADFVESK	Oxidation (M)
1	P02768	ALBU	Albumin	5.92	71317	1546	101	41.4	3	997.3505	2989.0297	2989.3321	-0.3024	0	59	SHCIAEVENDEMPADLPSLAADFVESK	Oxidation (M)
1	P02768	ALBU	Albumin	5.92	71317	1546	101	41.4	3	997.3549	2989.0429	2989.3321	-0.2892	0	41	SHCIAEVENDEMPADLPSLAADFVESK	Oxidation (M)
1	P02768	ALBU	Albumin	5.92	71317	1546	101	41.4	3	997.3768	2989.1086	2989.3321	-0.2235	0	45	SHCIAEVENDEMPADLPSLAADFVESK	Oxidation (M)

1	P02768	ALBU	Albumin	5.92	71317	1546	101	41.4	3	997.3875	2989.1407	2989.3321	-0.1914	0	35	SHCIAEVENDEMPADLPSLAADFVESK	Oxidation (M)
1	P02768	ALBU	Albumin	5.92	71317	1546	101	41.4	2	1495.605	2989.1954	2989.3321	-0.1366	0	39	SHCIAEVENDEMPADLPSLAADFVESK	Oxidation (M)
1	P02768	ALBU	Albumin	5.92	71317	1546	101	41.4	3	997.6552	2989.9438	2989.3321	0.6117	0	69	SHCIAEVENDEMPADLPSLAADFVESK	Oxidation (M)
2	P02790	HEMO	Hemopexin	6.555	52385	371	9	20.8	2	565.7516	1129.4886	1128.6444	0.8443	1	44	RLWWLDLK	
2	P02790	HEMO	Hemopexin	6.555	52385	371	9	20.8	2	571.9941	1141.9736	1141.6244	0.3493	0	54	QGHNSVFLIK	
2	P02790	HEMO	Hemopexin	6.555	52385	371	9	20.8	2	572.1501	1142.2856	1140.5815	1.7041	0	56	GGYTLVSGYPK	
2	P02790	HEMO	Hemopexin	6.555	52385	371	9	20.8	2	611.1481	1220.2816	1219.5986	0.6831	0	51	NFPSPVDAAFR	
2	P02790	HEMO	Hemopexin	6.555	52385	371	9	20.8	2	743.0792	1484.1438	1483.6806	0.4633	0	62	EWFWDLATGTMK	
2	P02790	HEMO	Hemopexin	6.555	52385	371	9	20.8	2	751.1636	1500.3126	1499.6755	0.6371	0	65	EWFWDLATGTMK	Oxidation (M)
2	P02790	HEMO	Hemopexin	6.555	52385	371	9	20.8	2	919.7122	1837.4098	1836.8795	0.5304	0	72	SGAQATWTELPWPHEK	
2	P02790	HEMO	Hemopexin	6.555	52385	371	9	20.8	3	613.5625	1837.6657	1836.8795	0.7862	0	55	SGAQATWTELPWPHEK	
2	P02790	HEMO	Hemopexin	6.555	52385	371	9	20.8	3	1073.984	3218.9302	3218.4635	0.4667	0	38	DGWHSWPIAHQWPQGPSAVDAAFSWEEK	
3	P81605	DCD	Dermcidin	6.08	11391	113	3	25.5	3	487.6609	1459.9609	1458.7566	1.2043	1	40	LGKDAVEDLESVGK	
3	P81605	DCD	Dermcidin	6.08	11391	113	3	25.5	2	731.2372	1460.4598	1458.7566	1.7033	1	58	LGKDAVEDLESVGK	

	3	P81605	DCD	Dermcidin	6.08	11391	113	3	25.5	2	734.3577	1466.7008	1465.7777	0.9232	1	54	GAVHDVKDVLDSVL
2670	1	P02768	ALBU	Albumin	5.92	71317	1310	53	37.8	1	695.457	694.4497	694.3286	0.1212	0	37	NYAEAK
	1	P02768	ALBU	Albumin	5.92	71317	1310	53	37.8	2	464.4876	926.9606	926.4861	0.4745	0	35	YLYEIAR
	1	P02768	ALBU	Albumin	5.92	71317	1310	53	37.8	1	940.607	939.5997	939.441	0.1587	0	46	DDNPNLPR
	1	P02768	ALBU	Albumin	5.92	71317	1310	53	37.8	2	471.0466	940.0786	939.441	0.6377	0	40	DDNPNLPR
	1	P02768	ALBU	Albumin	5.92	71317	1310	53	37.8	2	481.0756	960.1366	959.5552	0.5814	0	52	FQNALLVR
	1	P02768	ALBU	Albumin	5.92	71317	1310	53	37.8	2	481.2836	960.5526	959.5552	0.9974	0	46	FQNALLVR
	1	P02768	ALBU	Albumin	5.92	71317	1310	53	37.8	2	492.8766	983.7386	983.4811	0.2575	0	34	TYETTLEK
	1	P02768	ALBU	Albumin	5.92	71317	1310	53	37.8	2	500.9661	999.9176	999.5964	0.3212	0	50	QTALVELVK
	1	P02768	ALBU	Albumin	5.92	71317	1310	53	37.8	2	507.3026	1012.5906	1012.5917	-0.001	0	92	LVAASQAALGL
	1	P02768	ALBU	Albumin	5.92	71317	1310	53	37.8	2	507.5136	1013.0126	1012.5917	0.421	0	70	LVAASQAALGL
	1	P02768	ALBU	Albumin	5.92	71317	1310	53	37.8	1	1017.715	1016.7077	1016.5291	0.1786	0	46	SLHTLFGDK
	1	P02768	ALBU	Albumin	5.92	71317	1310	53	37.8	2	509.4196	1016.8246	1016.5291	0.2955	0	51	SLHTLFGDK
	1	P02768	ALBU	Albumin	5.92	71317	1310	53	37.8	2	528.5646	1055.1146	1054.5811	0.5336	1	50	KYLYEIAR

1	P02768	ALBU	Albumin	5.92	71317	1310	53	37.8	2	538.1537	1074.2928	1073.5353	0.7576	1	47	LDELRDEGK
1	P02768	ALBU	Albumin	5.92	71317	1310	53	37.8	2	565.0511	1128.0876	1127.6914	0.3963	1	60	KQTALVELVK
1	P02768	ALBU	Albumin	5.92	71317	1310	53	37.8	2	571.7357	1141.4568	1140.6866	0.7702	1	69	KLVAASQAALGL
1	P02768	ALBU	Albumin	5.92	71317	1310	53	37.8	2	575.4286	1148.8426	1148.6077	0.2349	0	54	LVNEVTEFAK
1	P02768	ALBU	Albumin	5.92	71317	1310	53	37.8	2	575.6702	1149.3258	1148.6077	0.7181	0	55	LVNEVTEFAK
1	P02768	ALBU	Albumin	5.92	71317	1310	53	37.8	2	614.0511	1226.0876	1225.5979	0.4898	1	48	FKDLGEENFK
1	P02768	ALBU	Albumin	5.92	71317	1310	53	37.8	2	614.6937	1227.3728	1225.5979	1.775	1	47	FKDLGEENFK
1	P02768	ALBU	Albumin	5.92	71317	1310	53	37.8	1	1311.741	1310.7337	1310.7347	-0.0009	0	42	HPDYSVVLLLR
1	P02768	ALBU	Albumin	5.92	71317	1310	53	37.8	2	656.6602	1311.3058	1310.7347	0.5712	0	51	HPDYSVVLLLR
1	P02768	ALBU	Albumin	5.92	71317	1310	53	37.8	2	671.7567	1341.4988	1341.6275	-0.1286	0	53	AVMDDFAAFVEK
1	P02768	ALBU	Albumin	5.92	71317	1310	53	37.8	1	1342.662	1341.6547	1341.6275	0.0272	0	44	AVMDDFAAFVEK
1	P02768	ALBU	Albumin	5.92	71317	1310	53	37.8	2	672.0452	1342.0758	1341.6275	0.4484	0	39	AVMDDFAAFVEK
1	P02768	ALBU	Albumin	5.92	71317	1310	53	37.8	2	672.2852	1342.5558	1341.6275	0.9284	0	61	AVMDDFAAFVEK
1	P02768	ALBU	Albumin	5.92	71317	1310	53	37.8	2	672.7336	1343.4526	1341.6275	1.8252	0	69	AVMDDFAAFVEK

1	P02768	ALBU	Albumin	5.92	71317	1310	53	37.8	2	679.8901	1357.7656	1357.6224	0.1432	0	75	AVMDDFAAFVEK	Oxidation (M)
1	P02768	ALBU	Albumin	5.92	71317	1310	53	37.8	2	680.1636	1358.3126	1357.6224	0.6902	0	38	AVMDDFAAFVEK	Oxidation (M)
1	P02768	ALBU	Albumin	5.92	71317	1310	53	37.8	3	490.1062	1467.2968	1466.8358	0.461	1	46	RHPDYSVVLLLR	
1	P02768	ALBU	Albumin	5.92	71317	1310	53	37.8	3	490.1232	1467.3478	1466.8358	0.512	1	49	RHPDYSVVLLLR	
1	P02768	ALBU	Albumin	5.92	71317	1310	53	37.8	3	490.1909	1467.5509	1466.8358	0.7151	1	37	RHPDYSVVLLLR	
1	P02768	ALBU	Albumin	5.92	71317	1310	53	37.8	2	734.9056	1467.7966	1466.8358	0.9609	1	36	RHPDYSVVLLLR	
1	P02768	ALBU	Albumin	5.92	71317	1310	53	37.8	3	490.3082	1467.9028	1466.8358	1.067	1	41	RHPDYSVVLLLR	
1	P02768	ALBU	Albumin	5.92	71317	1310	53	37.8	3	490.3312	1467.9718	1466.8358	1.136	1	35	RHPDYSVVLLLR	
1	P02768	ALBU	Albumin	5.92	71317	1310	53	37.8	3	490.3545	1468.0417	1466.8358	1.2059	1	55	RHPDYSVVLLLR	
1	P02768	ALBU	Albumin	5.92	71317	1310	53	37.8	3	490.5345	1468.5817	1466.8358	1.7459	1	46	RHPDYSVVLLLR	
1	P02768	ALBU	Albumin	5.92	71317	1310	53	37.8	2	756.6556	1511.2966	1510.8355	0.4611	0	75	VPQVSTPTLVEVSR	
1	P02768	ALBU	Albumin	5.92	71317	1310	53	37.8	3	504.8835	1511.6287	1510.8355	0.7931	0	38	VPQVSTPTLVEVSR	
1	P02768	ALBU	Albumin	5.92	71317	1310	53	37.8	2	812.6631	1623.3116	1622.7803	0.5314	0	67	DVFLGMFLYEYAR	
1	P02768	ALBU	Albumin	5.92	71317	1310	53	37.8	2	820.6682	1639.3218	1638.7752	0.5466	0	55	DVFLGMFLYEYAR	Oxidation (M)

1	P02768	ALBU	Albumin	5.92	71317	1310	53	37.8	2	820.8547	1639.6948	1638.9305	0.7644	1	82	KVPQVSTPTLVEVSR	
1	P02768	ALBU	Albumin	5.92	71317	1310	53	37.8	3	547.7425	1640.2057	1638.9305	1.2752	1	37	KVPQVSTPTLVEVSR	
1	P02768	ALBU	Albumin	5.92	71317	1310	53	37.8	3	547.8025	1640.3857	1638.9305	1.4552	1	66	KVPQVSTPTLVEVSR	
1	P02768	ALBU	Albumin	5.92	71317	1310	53	37.8	2	872.2662	1742.5178	1741.8868	0.6311	0	44	HPYFYAPELLFFAK	
1	P02768	ALBU	Albumin	5.92	71317	1310	53	37.8	3	582.2422	1743.7048	1741.8868	1.818	0	53	HPYFYAPELLFFAK	
1	P02768	ALBU	Albumin	5.92	71317	1310	53	37.8	3	682.7285	2045.1637	2044.0881	1.0756	0	37	VFDEFKPLVEEPQNLIK	
1	P02768	ALBU	Albumin	5.92	71317	1310	53	37.8	3	682.8611	2045.5615	2044.0881	1.4734	0	43	VFDEFKPLVEEPQNLIK	
1	P02768	ALBU	Albumin	5.92	71317	1310	53	37.8	2	1023.933	2045.8514	2044.0881	1.7634	0	43	VFDEFKPLVEEPQNLIK	
1	P02768	ALBU	Albumin	5.92	71317	1310	53	37.8	2	1333.095	2664.1754	2665.2516	-1.0762	0	46	LVRPEVDVMCTAFHDNEETFLK	Oxidation (M)
1	P02768	ALBU	Albumin	5.92	71317	1310	53	37.8	3	889.5762	2665.7068	2665.2516	0.4551	0	46	LVRPEVDVMCTAFHDNEETFLK	Oxidation (M)
1	P02768	ALBU	Albumin	5.92	71317	1310	53	37.8	3	997.3549	2989.0429	2989.3321	-0.2892	0	56	SHCIAEVENDEMPADLPSLAADFVESK	Oxidation (M)
1	P02768	ALBU	Albumin	5.92	71317	1310	53	37.8	3	997.6742	2990.0008	2989.3321	0.6687	0	58	SHCIAEVENDEMPADLPSLAADFVESK	Oxidation (M)
2	P02790	HEMO	Hemopexin	6.55	52385	1011	2	6.1	2	743.0872	1484.1598	1483.6806	0.4793	0	56	EWFWDLATGTMK	
2	P02790	HEMO	Hemopexin	6.55	52385	1011	2	6.1	3	613.5508	1837.6306	1836.8795	0.7511	0	45	SGAQATWTELPWPHEK	

2703	1	P02768	ALBU	Albumin	5.92	71317	1521	127	40.2	1	695.45	694.4427	694.3286	0.1142	0	40	NYAEAK
	1	P02768	ALBU	Albumin	5.92	71317	1521	127	40.2	1	789.497	788.4897	788.4644	0.0254	0	36	LVTDLTK
	1	P02768	ALBU	Albumin	5.92	71317	1521	127	40.2	1	880.375	879.3677	879.4338	-0.0661	0	44	AEFAEVSK
	1	P02768	ALBU	Albumin	5.92	71317	1521	127	40.2	2	464.6111	927.2076	926.4861	0.7215	0	35	YLYEIAR
	1	P02768	ALBU	Albumin	5.92	71317	1521	127	40.2	2	464.8316	927.6486	926.4861	1.1625	0	35	YLYEIAR
	1	P02768	ALBU	Albumin	5.92	71317	1521	127	40.2	1	940.553	939.5457	939.441	0.1047	0	39	DDNPNLPR
	1	P02768	ALBU	Albumin	5.92	71317	1521	127	40.2	1	951.522	950.5147	950.4345	0.0802	0	45	DLGEENFK
	1	P02768	ALBU	Albumin	5.92	71317	1521	127	40.2	2	476.5371	951.0596	950.4345	0.6251	0	35	DLGEENFK
	1	P02768	ALBU	Albumin	5.92	71317	1521	127	40.2	2	480.1186	958.2226	959.5552	-1.3326	0	52	FQNALLVR
	1	P02768	ALBU	Albumin	5.92	71317	1521	127	40.2	2	481.0786	960.1426	959.5552	0.5874	0	47	FQNALLVR
	1	P02768	ALBU	Albumin	5.92	71317	1521	127	40.2	2	481.1466	960.2786	959.5552	0.7234	0	47	FQNALLVR
	1	P02768	ALBU	Albumin	5.92	71317	1521	127	40.2	2	481.3181	960.6216	959.5552	1.0664	0	40	FQNALLVR
	1	P02768	ALBU	Albumin	5.92	71317	1521	127	40.2	2	492.9786	983.9426	983.4811	0.4615	0	36	TYETTLEK
	1	P02768	ALBU	Albumin	5.92	71317	1521	127	40.2	2	493.1296	984.2446	983.4811	0.7635	0	34	TYETTLEK

1	P02768	ALBU	Albumin	5.92	71317	1521	127	40.2	2	501.0541	1000.0936	999.5964	0.4972	0	51	QTALVELVK
1	P02768	ALBU	Albumin	5.92	71317	1521	127	40.2	2	501.2386	1000.4626	999.5964	0.8662	0	58	QTALVELVK
1	P02768	ALBU	Albumin	5.92	71317	1521	127	40.2	2	507.5861	1013.1576	1012.5917	0.566	0	91	LVAASQAALGL
1	P02768	ALBU	Albumin	5.92	71317	1521	127	40.2	1	1017.585	1016.5777	1016.5291	0.0486	0	50	SLHTLFGDK
1	P02768	ALBU	Albumin	5.92	71317	1521	127	40.2	2	509.4576	1016.9006	1016.5291	0.3715	0	45	SLHTLFGDK
1	P02768	ALBU	Albumin	5.92	71317	1521	127	40.2	2	509.5286	1017.0426	1016.5291	0.5135	0	38	SLHTLFGDK
1	P02768	ALBU	Albumin	5.92	71317	1521	127	40.2	2	509.5791	1017.1436	1016.5291	0.6145	0	45	SLHTLFGDK
1	P02768	ALBU	Albumin	5.92	71317	1521	127	40.2	2	528.7742	1055.5338	1054.5811	0.9528	1	49	KYLYEIAR
1	P02768	ALBU	Albumin	5.92	71317	1521	127	40.2	2	538.1111	1074.2076	1073.5353	0.6724	1	37	LDELRDEGK
1	P02768	ALBU	Albumin	5.92	71317	1521	127	40.2	2	538.1426	1074.2706	1073.5353	0.7354	1	41	LDELRDEGK
1	P02768	ALBU	Albumin	5.92	71317	1521	127	40.2	1	1128.611	1127.6037	1127.6914	-0.0877	1	54	KQTALVELVK
1	P02768	ALBU	Albumin	5.92	71317	1521	127	40.2	2	565.1921	1128.3696	1127.6914	0.6783	1	46	KQTALVELVK
1	P02768	ALBU	Albumin	5.92	71317	1521	127	40.2	1	1141.628	1140.6207	1140.6866	-0.0659	1	39	KLVAASQAALGL
1	P02768	ALBU	Albumin	5.92	71317	1521	127	40.2	2	571.6857	1141.3568	1140.6866	0.6702	1	63	KLVAASQAALGL

1	P02768	ALBU	Albumin	5.92	71317	1521	127	40.2	1	1148.98	1147.9727	1148.6077	-0.635	0	38	LVNEVTEFAK
1	P02768	ALBU	Albumin	5.92	71317	1521	127	40.2	2	575.0671	1148.1196	1148.6077	-0.4881	0	52	LVNEVTEFAK
1	P02768	ALBU	Albumin	5.92	71317	1521	127	40.2	2	575.2831	1148.5516	1148.6077	-0.0561	0	48	LVNEVTEFAK
1	P02768	ALBU	Albumin	5.92	71317	1521	127	40.2	2	575.3171	1148.6196	1148.6077	0.0119	0	42	LVNEVTEFAK
1	P02768	ALBU	Albumin	5.92	71317	1521	127	40.2	2	575.4236	1148.8326	1148.6077	0.2249	0	48	LVNEVTEFAK
1	P02768	ALBU	Albumin	5.92	71317	1521	127	40.2	2	575.5276	1149.0406	1148.6077	0.4329	0	50	LVNEVTEFAK
1	P02768	ALBU	Albumin	5.92	71317	1521	127	40.2	2	575.6432	1149.2718	1148.6077	0.6641	0	55	LVNEVTEFAK
1	P02768	ALBU	Albumin	5.92	71317	1521	127	40.2	2	575.9582	1149.9018	1148.6077	1.2941	0	41	LVNEVTEFAK
1	P02768	ALBU	Albumin	5.92	71317	1521	127	40.2	2	576.2366	1150.4586	1148.6077	1.8509	0	59	LVNEVTEFAK
1	P02768	ALBU	Albumin	5.92	71317	1521	127	40.2	2	614.4097	1226.8048	1225.5979	1.207	1	42	FKDLGEENFK
1	P02768	ALBU	Albumin	5.92	71317	1521	127	40.2	2	614.6467	1227.2788	1225.5979	1.681	1	47	FKDLGEENFK
1	P02768	ALBU	Albumin	5.92	71317	1521	127	40.2	2	656.7407	1311.4668	1310.7347	0.7322	0	59	HPDYSVVLLLR
1	P02768	ALBU	Albumin	5.92	71317	1521	127	40.2	2	656.9241	1311.8336	1310.7347	1.099	0	57	HPDYSVVLLLR
1	P02768	ALBU	Albumin	5.92	71317	1521	127	40.2	1	1312.884	1311.8767	1310.7347	1.1421	0	39	HPDYSVVLLLR

1	P02768	ALBU	Albumin	5.92	71317	1521	127	40.2	1	1342.642	1341.6347	1341.6275	0.0072	0	51	AVMDDFAAFVEK	
1	P02768	ALBU	Albumin	5.92	71317	1521	127	40.2	2	671.8367	1341.6588	1341.6275	0.0314	0	76	AVMDDFAAFVEK	
1	P02768	ALBU	Albumin	5.92	71317	1521	127	40.2	2	671.9996	1341.9846	1341.6275	0.3572	0	38	AVMDDFAAFVEK	
1	P02768	ALBU	Albumin	5.92	71317	1521	127	40.2	2	672.0452	1342.0758	1341.6275	0.4484	0	44	AVMDDFAAFVEK	
1	P02768	ALBU	Albumin	5.92	71317	1521	127	40.2	2	672.0467	1342.0788	1341.6275	0.4514	0	68	AVMDDFAAFVEK	
1	P02768	ALBU	Albumin	5.92	71317	1521	127	40.2	2	672.1971	1342.3796	1341.6275	0.7522	0	48	AVMDDFAAFVEK	
1	P02768	ALBU	Albumin	5.92	71317	1521	127	40.2	2	672.2162	1342.4178	1341.6275	0.7904	0	62	AVMDDFAAFVEK	
1	P02768	ALBU	Albumin	5.92	71317	1521	127	40.2	2	672.4582	1342.9018	1341.6275	1.2744	0	42	AVMDDFAAFVEK	
1	P02768	ALBU	Albumin	5.92	71317	1521	127	40.2	2	672.4962	1342.9778	1341.6275	1.3504	0	61	AVMDDFAAFVEK	
1	P02768	ALBU	Albumin	5.92	71317	1521	127	40.2	2	672.6682	1343.3218	1341.6275	1.6944	0	39	AVMDDFAAFVEK	
1	P02768	ALBU	Albumin	5.92	71317	1521	127	40.2	2	672.6946	1343.3746	1341.6275	1.7472	0	57	AVMDDFAAFVEK	
1	P02768	ALBU	Albumin	5.92	71317	1521	127	40.2	1	1358.711	1357.7037	1357.6224	0.0813	0	41	AVMDDFAAFVEK	Oxidation (M)
1	P02768	ALBU	Albumin	5.92	71317	1521	127	40.2	2	680.1531	1358.2916	1357.6224	0.6692	0	68	AVMDDFAAFVEK	Oxidation (M)
1	P02768	ALBU	Albumin	5.92	71317	1521	127	40.2	3	489.9062	1466.6968	1466.8358	-0.139	1	55	RHPDYSVVLLLR	

1	P02768	ALBU	Albumin	5.92	71317	1521	127	40.2	3	490.1195	1467.3367	1466.8358	0.50009	1	40	RHPDYSVVLLLR
1	P02768	ALBU	Albumin	5.92	71317	1521	127	40.2	2	734.7577	1467.5008	1466.8358	0.6651	1	50	RHPDYSVVLLLR
1	P02768	ALBU	Albumin	5.92	71317	1521	127	40.2	3	490.1872	1467.5398	1466.8358	0.704	1	39	RHPDYSVVLLLR
1	P02768	ALBU	Albumin	5.92	71317	1521	127	40.2	3	490.1978	1467.5716	1466.8358	0.7358	1	53	RHPDYSVVLLLR
1	P02768	ALBU	Albumin	5.92	71317	1521	127	40.2	3	490.2052	1467.5938	1466.8358	0.758	1	48	RHPDYSVVLLLR
1	P02768	ALBU	Albumin	5.92	71317	1521	127	40.2	3	490.2188	1467.6346	1466.8358	0.7988	1	47	RHPDYSVVLLLR
1	P02768	ALBU	Albumin	5.92	71317	1521	127	40.2	3	490.2525	1467.7357	1466.8358	0.8999	1	49	RHPDYSVVLLLR
1	P02768	ALBU	Albumin	5.92	71317	1521	127	40.2	3	490.2622	1467.7648	1466.8358	0.929	1	40	RHPDYSVVLLLR
1	P02768	ALBU	Albumin	5.92	71317	1521	127	40.2	1	1468.82	1467.8127	1466.8358	0.977	1	41	RHPDYSVVLLLR
1	P02768	ALBU	Albumin	5.92	71317	1521	127	40.2	3	490.3159	1467.9259	1466.8358	1.0901	1	47	RHPDYSVVLLLR
1	P02768	ALBU	Albumin	5.92	71317	1521	127	40.2	3	490.3925	1468.1557	1466.8358	1.3199	1	50	RHPDYSVVLLLR
1	P02768	ALBU	Albumin	5.92	71317	1521	127	40.2	3	490.4085	1468.2037	1466.8358	1.3679	1	36	RHPDYSVVLLLR
1	P02768	ALBU	Albumin	5.92	71317	1521	127	40.2	3	490.4229	1468.2469	1466.8358	1.4111	1	52	RHPDYSVVLLLR
1	P02768	ALBU	Albumin	5.92	71317	1521	127	40.2	3	490.5252	1468.5538	1466.8358	1.718	1	48	RHPDYSVVLLLR

1	P02768	ALBU	Albumin	5.92	71317	1521	127	40.2	2	756.7607	1511.5068	1510.8355	0.6713	0	52	VPQVSTPTLVEVSR	
1	P02768	ALBU	Albumin	5.92	71317	1521	127	40.2	3	504.8479	1511.5219	1510.8355	0.6863	0	35	VPQVSTPTLVEVSR	
1	P02768	ALBU	Albumin	5.92	71317	1521	127	40.2	2	756.8937	1511.7728	1510.8355	0.9373	0	58	VPQVSTPTLVEVSR	
1	P02768	ALBU	Albumin	5.92	71317	1521	127	40.2	2	812.3066	1622.5986	1622.7803	-0.1816	0	73	DVFLGMFLYEYAR	
1	P02768	ALBU	Albumin	5.92	71317	1521	127	40.2	2	812.6686	1623.3226	1622.7803	0.5424	0	68	DVFLGMFLYEYAR	
1	P02768	ALBU	Albumin	5.92	71317	1521	127	40.2	2	812.7032	1623.3918	1622.7803	0.6116	0	70	DVFLGMFLYEYAR	
1	P02768	ALBU	Albumin	5.92	71317	1521	127	40.2	2	812.7542	1623.4938	1622.7803	0.7136	0	47	DVFLGMFLYEYAR	
1	P02768	ALBU	Albumin	5.92	71317	1521	127	40.2	2	812.7596	1623.5046	1622.7803	0.7244	0	49	DVFLGMFLYEYAR	
1	P02768	ALBU	Albumin	5.92	71317	1521	127	40.2	1	1624.721	1623.7137	1622.7803	0.9334	0	57	DVFLGMFLYEYAR	
1	P02768	ALBU	Albumin	5.92	71317	1521	127	40.2	2	812.9446	1623.8746	1622.7803	1.0944	0	47	DVFLGMFLYEYAR	
1	P02768	ALBU	Albumin	5.92	71317	1521	127	40.2	2	812.9842	1623.9538	1622.7803	1.1736	0	50	DVFLGMFLYEYAR	
1	P02768	ALBU	Albumin	5.92	71317	1521	127	40.2	2	820.6782	1639.3418	1638.9305	0.4114	1	82	KVPQVSTPTLVEVSR	
1	P02768	ALBU	Albumin	5.92	71317	1521	127	40.2	2	820.6962	1639.3778	1638.9305	0.4474	1	34	KVPQVSTPTLVEVSR	
1	P02768	ALBU	Albumin	5.92	71317	1521	127	40.2	2	820.7217	1639.4288	1638.7752	0.6536	0	55	DVFLGMFLYEYAR	Oxidation (M)

1	P02768	ALBU	Albumin	5.92	71317	1521	127	40.2	2	820.8217	1639.6288	1638.7752	0.8536	0	50	DVFLGMFLYEYAR	Oxidation (M)
1	P02768	ALBU	Albumin	5.92	71317	1521	127	40.2	2	820.8782	1639.7418	1638.9305	0.8114	1	94	KVPQVSTPTLVEVSR	
1	P02768	ALBU	Albumin	5.92	71317	1521	127	40.2	3	547.5985	1639.7737	1638.9305	0.8432	1	41	KVPQVSTPTLVEVSR	
1	P02768	ALBU	Albumin	5.92	71317	1521	127	40.2	3	547.7222	1640.1448	1638.9305	1.2143	1	41	KVPQVSTPTLVEVSR	
1	P02768	ALBU	Albumin	5.92	71317	1521	127	40.2	3	547.7965	1640.3677	1638.9305	1.4372	1	60	KVPQVSTPTLVEVSR	
1	P02768	ALBU	Albumin	5.92	71317	1521	127	40.2	3	547.8091	1640.4055	1638.9305	1.475	1	67	KVPQVSTPTLVEVSR	
1	P02768	ALBU	Albumin	5.92	71317	1521	127	40.2	3	547.8149	1640.4229	1638.9305	1.4924	1	49	KVPQVSTPTLVEVSR	
1	P02768	ALBU	Albumin	5.92	71317	1521	127	40.2	3	547.8608	1640.5606	1638.9305	1.6301	1	41	KVPQVSTPTLVEVSR	
1	P02768	ALBU	Albumin	5.92	71317	1521	127	40.2	2	821.3602	1640.7058	1638.9305	1.7754	1	49	KVPQVSTPTLVEVSR	
1	P02768	ALBU	Albumin	5.92	71317	1521	127	40.2	3	547.9539	1640.8399	1638.9305	1.9094	1	43	KVPQVSTPTLVEVSR	
1	P02768	ALBU	Albumin	5.92	71317	1521	127	40.2	1	1742.833	1741.8257	1741.8868	-0.0611	0	57	HPYFYAPELLFFAK	
1	P02768	ALBU	Albumin	5.92	71317	1521	127	40.2	2	872.3477	1742.6808	1741.8868	0.7941	0	44	HPYFYAPELLFFAK	
1	P02768	ALBU	Albumin	5.92	71317	1521	127	40.2	3	582.0098	1743.0076	1741.8868	1.1208	0	56	HPYFYAPELLFFAK	
1	P02768	ALBU	Albumin	5.92	71317	1521	127	40.2	3	681.9518	2042.8336	2044.0881	-1.2545	0	34	VFDEFKPLVEEPQNLIK	

1	P02768	ALBU	Albumin	5.92	71317	1521	127	40.2	3	682.2358	2043.6856	2044.0881	-0.4025	0	44	VFDEFKPLVEEPQNLIK	
1	P02768	ALBU	Albumin	5.92	71317	1521	127	40.2	3	682.4755	2044.4047	2044.0881	0.3166	0	41	VFDEFKPLVEEPQNLIK	
1	P02768	ALBU	Albumin	5.92	71317	1521	127	40.2	3	682.4819	2044.4239	2044.0881	0.3358	0	37	VFDEFKPLVEEPQNLIK	
1	P02768	ALBU	Albumin	5.92	71317	1521	127	40.2	3	682.5228	2044.5466	2044.0881	0.4585	0	39	VFDEFKPLVEEPQNLIK	
1	P02768	ALBU	Albumin	5.92	71317	1521	127	40.2	2	1023.491	2044.9674	2044.0881	0.8794	0	35	VFDEFKPLVEEPQNLIK	
1	P02768	ALBU	Albumin	5.92	71317	1521	127	40.2	2	1023.538	2045.0614	2044.0881	0.9734	0	50	VFDEFKPLVEEPQNLIK	
1	P02768	ALBU	Albumin	5.92	71317	1521	127	40.2	3	682.7945	2045.3617	2044.0881	1.2736	0	42	VFDEFKPLVEEPQNLIK	
1	P02768	ALBU	Albumin	5.92	71317	1521	127	40.2	3	682.8002	2045.3788	2044.0881	1.2907	0	50	VFDEFKPLVEEPQNLIK	
1	P02768	ALBU	Albumin	5.92	71317	1521	127	40.2	3	682.8225	2045.4457	2044.0881	1.3576	0	36	VFDEFKPLVEEPQNLIK	
1	P02768	ALBU	Albumin	5.92	71317	1521	127	40.2	3	682.8328	2045.4766	2044.0881	1.3885	0	44	VFDEFKPLVEEPQNLIK	
1	P02768	ALBU	Albumin	5.92	71317	1521	127	40.2	2	1023.978	2045.9414	2044.0881	1.8534	0	53	VFDEFKPLVEEPQNLIK	
1	P02768	ALBU	Albumin	5.92	71317	1521	127	40.2	3	888.8355	2663.4847	2665.2516	-1.767	0	38	LVRPEVDVMCTAFHDNEETFLK	Oxidation (M)
1	P02768	ALBU	Albumin	5.92	71317	1521	127	40.2	3	889.1192	2664.3358	2665.2516	-0.9159	0	56	LVRPEVDVMCTAFHDNEETFLK	Oxidation (M)
1	P02768	ALBU	Albumin	5.92	71317	1521	127	40.2	3	889.2405	2664.6997	2665.2516	-0.552	0	56	LVRPEVDVMCTAFHDNEETFLK	Oxidation (M)

1	P02768	ALBU	Albumin	5.92	71317	1521	127	40.2	3	889.4498	2665.3276	2665.2516	0.0759	0	35	LVRPEVDVMCTAFHDNEETFLK	Oxidation (M)
1	P02768	ALBU	Albumin	5.92	71317	1521	127	40.2	3	889.4608	2665.3606	2665.2516	0.1089	0	52	LVRPEVDVMCTAFHDNEETFLK	Oxidation (M)
1	P02768	ALBU	Albumin	5.92	71317	1521	127	40.2	2	1333.95	2665.8854	2665.2516	0.6338	0	56	LVRPEVDVMCTAFHDNEETFLK	Oxidation (M)
1	P02768	ALBU	Albumin	5.92	71317	1521	127	40.2	3	931.9412	2792.8018	2793.3466	-0.5448	1	59	LVRPEVDVMCTAFHDNEETFLKK	Oxidation (M)
1	P02768	ALBU	Albumin	5.92	71317	1521	127	40.2	3	997.1422	2988.4048	2989.3321	-0.9273	0	36	SHCIAEVENDEMPADLPSLAADFVESK	Oxidation (M)
1	P02768	ALBU	Albumin	5.92	71317	1521	127	40.2	3	997.1798	2988.5176	2989.3321	-0.8145	0	66	SHCIAEVENDEMPADLPSLAADFVESK	Oxidation (M)
1	P02768	ALBU	Albumin	5.92	71317	1521	127	40.2	3	997.2462	2988.7168	2989.3321	-0.6153	0	44	SHCIAEVENDEMPADLPSLAADFVESK	Oxidation (M)
1	P02768	ALBU	Albumin	5.92	71317	1521	127	40.2	3	997.2752	2988.8038	2989.3321	-0.5283	0	46	SHCIAEVENDEMPADLPSLAADFVESK	Oxidation (M)
1	P02768	ALBU	Albumin	5.92	71317	1521	127	40.2	3	997.4799	2989.4179	2989.3321	0.0858	0	53	SHCIAEVENDEMPADLPSLAADFVESK	Oxidation (M)
1	P02768	ALBU	Albumin	5.92	71317	1521	127	40.2	3	997.5042	2989.4908	2989.3321	0.1587	0	40	SHCIAEVENDEMPADLPSLAADFVESK	Oxidation (M)
1	P02768	ALBU	Albumin	5.92	71317	1521	127	40.2	3	997.5298	2989.5676	2989.3321	0.2355	0	52	SHCIAEVENDEMPADLPSLAADFVESK	Oxidation (M)
1	P02768	ALBU	Albumin	5.92	71317	1521	127	40.2	2	1496.031	2990.0474	2989.3321	0.7154	0	40	SHCIAEVENDEMPADLPSLAADFVESK	Oxidation (M)
1	P02768	ALBU	Albumin	5.92	71317	1521	127	40.2	3	997.7391	2990.1955	2989.3321	0.8634	0	52	SHCIAEVENDEMPADLPSLAADFVESK	Oxidation (M)
1	P02768	ALBU	Albumin	5.92	71317	1521	127	40.2	3	997.7995	2990.3767	2989.3321	1.0446	0	45	SHCIAEVENDEMPADLPSLAADFVESK	Oxidation (M)

	1	P02768	ALBU	Albumin	5.92	71317	1521	127	40.2	3	997.8405	2990.4997	2989.3321	1.1676	0	39	SHCIAEVENDEMPADLPSLAADFVESK	Oxidation (M)
2715	1	P02768	ALBU	Albumin	5.92	71317	1096	31	36.8	1	789.65	788.6427	788.4644	0.1784	0	36	LVTDLTK	
	1	P02768	ALBU	Albumin	5.92	71317	1096	31	36.8	2	471.1861	940.3576	939.441	0.9167	0	40	DDNPNLPR	
	1	P02768	ALBU	Albumin	5.92	71317	1096	31	36.8	2	481.1031	960.1916	959.5552	0.6364	0	50	FQNALLVR	
	1	P02768	ALBU	Albumin	5.92	71317	1096	31	36.8	2	493.1316	984.2486	983.4811	0.7675	0	34	TYETTLEK	
	1	P02768	ALBU	Albumin	5.92	71317	1096	31	36.8	2	501.0526	1000.0906	999.5964	0.4942	0	61	QTALVELVK	
	1	P02768	ALBU	Albumin	5.92	71317	1096	31	36.8	2	507.5326	1013.0506	1012.5917	0.459	0	95	LVAASQAALGL	
	1	P02768	ALBU	Albumin	5.92	71317	1096	31	36.8	1	1017.806	1016.7987	1016.5291	0.2696	0	36	SLHTLFGDK	
	1	P02768	ALBU	Albumin	5.92	71317	1096	31	36.8	2	538.0632	1074.1118	1073.5353	0.5766	1	34	LDELRDEGK	
	1	P02768	ALBU	Albumin	5.92	71317	1096	31	36.8	2	565.2751	1128.5356	1127.6914	0.8443	1	37	KQTALVELVK	
	1	P02768	ALBU	Albumin	5.92	71317	1096	31	36.8	2	571.6617	1141.3088	1140.6866	0.6222	1	44	KLVAASQAALGL	
	1	P02768	ALBU	Albumin	5.92	71317	1096	31	36.8	2	575.6556	1149.2966	1148.6077	0.6889	0	51	LVNEVTEFAK	
	1	P02768	ALBU	Albumin	5.92	71317	1096	31	36.8	2	614.2587	1226.5028	1225.5979	0.905	1	35	FKDLGEENFK	
	1	P02768	ALBU	Albumin	5.92	71317	1096	31	36.8	2	656.4207	1310.8268	1310.7347	0.0922	0	55	HPDYSVVLLLR	

1	P02768	ALBU	Albumin	5.92	71317	1096	31	36.8	2	672.6122	1343.2098	1341.6275	1.5824	0	69	AVMDDFAAFVEK	
1	P02768	ALBU	Albumin	5.92	71317	1096	31	36.8	2	680.3087	1358.6028	1357.6224	0.9804	0	73	AVMDDFAAFVEK	Oxidation (M)
1	P02768	ALBU	Albumin	5.92	71317	1096	31	36.8	2	734.7032	1467.3918	1466.8358	0.5561	1	40	RHPDYSVVLLLR	
1	P02768	ALBU	Albumin	5.92	71317	1096	31	36.8	3	490.2458	1467.7156	1466.8358	0.8798	1	50	RHPDYSVVLLLR	
1	P02768	ALBU	Albumin	5.92	71317	1096	31	36.8	2	812.8917	1623.7688	1622.7803	0.9886	0	48	DVFLGMFLYEYAR	
1	P02768	ALBU	Albumin	5.92	71317	1096	31	36.8	2	820.6791	1639.3436	1638.7752	0.5684	0	70	DVFLGMFLYEYAR	Oxidation (M)
1	P02768	ALBU	Albumin	5.92	71317	1096	31	36.8	3	547.5702	1639.6888	1638.9305	0.7583	1	68	KVPQVSTPTLVEVSR	
1	P02768	ALBU	Albumin	5.92	71317	1096	31	36.8	2	821.0977	1640.1808	1638.9305	1.2504	1	77	KVPQVSTPTLVEVSR	
1	P02768	ALBU	Albumin	5.92	71317	1096	31	36.8	3	582.1862	1743.5368	1741.8868	1.65	0	48	HPYFYAPELLFFAK	
1	P02768	ALBU	Albumin	5.92	71317	1096	31	36.8	2	872.8611	1743.7076	1741.8868	1.8209	0	42	HPYFYAPELLFFAK	
1	P02768	ALBU	Albumin	5.92	71317	1096	31	36.8	3	633.8625	1898.5657	1897.9879	0.5778	1	56	RHPYFYAPELLFFAK	
1	P02768	ALBU	Albumin	5.92	71317	1096	31	36.8	2	1023.655	2045.2954	2044.0881	1.2074	0	55	VFDEFKPLVEEPQNLIK	
1	P02768	ALBU	Albumin	5.92	71317	1096	31	36.8	3	682.8088	2045.4046	2044.0881	1.3165	0	42	VFDEFKPLVEEPQNLIK	
1	P02768	ALBU	Albumin	5.92	71317	1096	31	36.8	2	1332.895	2663.7754	2665.2516	-1.4762	0	38	LVRPEVDVMCTAFHDNEETFLK	Oxidation (M)

1	P02768	ALBU	Albumin	5.92	71317	1096	31	36.8	3	889.2375	2664.6907	2665.2516	-0.561	0	34	LVRPEVDVMCTAFHDNEETFLK	Oxidation (M)
1	P02768	ALBU	Albumin	5.92	71317	1096	31	36.8	3	889.4345	2665.2817	2665.2516	0.03	0	46	LVRPEVDVMCTAFHDNEETFLK	Oxidation (M)
1	P02768	ALBU	Albumin	5.92	71317	1096	31	36.8	3	997.0718	2988.1936	2989.3321	-1.1385	0	34	SHCIAEVENDEMPADLPSLAADFVESK	Oxidation (M)
1	P02768	ALBU	Albumin	5.92	71317	1096	31	36.8	3	997.5815	2989.7227	2989.3321	0.3906	0	37	SHCIAEVENDEMPADLPSLAADFVESK	Oxidation (M)
2	P01876	IGHA1	Ig alpha-1 chain C region	6.08	38486	321	6	22.9	2	466.5726	931.1306	930.5386	0.5921	0	40	TPLTATLSK	
2	P01876	IGHA1	Ig alpha-1 chain C region	6.08	38486	321	6	22.9	2	470.8756	939.7366	939.4774	0.2593	0	43	SAVQGPPER	
2	P01876	IGHA1	Ig alpha-1 chain C region	6.08	38486	321	6	22.9	2	607.5057	1212.9968	1212.6251	0.3717	0	55	WLQGSQELPR	
2	P01876	IGHA1	Ig alpha-1 chain C region	6.08	38486	321	6	22.9	2	771.7097	1541.4048	1539.7206	1.6843	0	75	DASGVTFTWTPSSGK	
2	P01876	IGHA1	Ig alpha-1 chain C region	6.08	38486	321	6	22.9	3	613.1891	1836.5455	1834.9425	1.603	0	60	QEPSQGTTTFAVTSILR	
2	P01876	IGHA1	Ig alpha-1 chain C region	6.08	38486	321	6	22.9	3	784.8948	2351.6626	2352.1243	-0.4617	1	48	KGDTFSCMVGHEALPLAFTQK	Oxidation (M)
3	P01877	IGHA2	Ig alpha-2 chain C region	5.71	37301	263	5	21.2	2	470.8756	939.7366	939.4774	0.2593	0	43	SAVQGPPER	
3	P01877	IGHA	Ig alpha-2	5.7	3730	263	5	21.2	2	607.505	1212.996	1212.625	0.371	0	55	WLQGSQELPR	

		2	chain C region	1	1					7	8	1	7				
3	P01877	IGHA2	Ig alpha-2 chain C region	5.71	37301	263	5	21.2	2	757.3212	1512.6278	1511.6893	0.9386	0	58	DASGATFTWTPSSGK	
3	P01877	IGHA2	Ig alpha-2 chain C region	5.71	37301	263	5	21.2	3	613.1891	1836.5455	1834.9425	1.603	0	60	QEPSQGTTTFAVTSILR	
3	P01877	IGHA2	Ig alpha-2 chain C region	5.71	37301	263	5	21.2	3	784.8948	2351.6626	2352.1243	-0.4617	1	48	KGDTFSCMVGHEALPLAFTQK	Oxidation (M)
4	P04264	K2C1	Keratin, type II cytoskeletal 1	8.15	66170	258	4	6.8	2	533.5737	1065.1328	1064.5138	0.619	0	44	AQYEDIAQK	
4	P04264	K2C1	Keratin, type II cytoskeletal 1	8.15	66170	258	4	6.8	2	633.6907	1265.3668	1264.6299	0.7369	0	49	TNAENEFVTIK	
4	P04264	K2C1	Keratin, type II cytoskeletal 1	8.15	66170	258	4	6.8	2	679.4572	1356.8998	1356.6885	0.2114	0	79	LNDLEDALQQAK	
4	P04264	K2C1	Keratin, type II cytoskeletal 1	8.15	66170	258	4	6.8	2	738.7327	1475.4508	1474.778	0.6729	0	94	FLEQQNQVLQTK	
5	P01766	HV305	Ig heavy chain V-III region BRO	6.45	13332	119	3	15.8	2	941.8242	1881.6338	1880.9956	0.6382	0	59	EVQLVESGGGLVQPGGSLR	
5	P01766	HV305	Ig heavy chain V-III region BRO	6.45	13332	119	3	15.8	3	628.4485	1882.3237	1880.9956	1.3281	0	84	EVQLVESGGGLVQPGGSLR	
5	P01766	HV30	Ig heavy chain	6.4	1333	119	3	15.8	2	942.320	1882.626	1880.995	1.631	0	119	EVQLVESGGGLVQPGGSLR	

			5	V-III region BRO	5	2					7	8	6	2			
	5	P01777	HV316	Ig heavy chain V-III region TEI	8.72	12908	119	3	16.0	2	941.8242	1881.6338	1880.9956	0.6382	0	59	EVQLVESGGGLVQPGGSLR
	5	P01777	HV316	Ig heavy chain V-III region TEI	8.72	12908	119	3	16.0	3	628.4485	1882.3237	1880.9956	1.3281	0	84	EVQLVESGGGLVQPGGSLR
	5	P01777	HV316	Ig heavy chain V-III region TEI	8.72	12908	119	3	16.0	2	942.3207	1882.6268	1880.9956	1.6312	0	119	EVQLVESGGGLVQPGGSLR
	6	P01765	HV304	Ig heavy chain V-III region TIL	9.24	12462	68	1	16.5	2	948.9042	1895.7938	1895.0112	0.7826	0	68	EVQLLESGGGLVQPGGSLR
	6	P01774	HV313	Ig heavy chain V-III region POM	8.05	13059	68	1	16.0	2	948.9042	1895.7938	1895.0112	0.7826	0	68	EVQLLESGGGLVQPGGSLR
	6	P01776	HV315	Ig heavy chain V-III region WAS	8.07	13196	68	1	16.2	2	948.9042	1895.7938	1895.0112	0.7826	0	68	EVQLLESGGGLVQPGGSLR
	6	P01779	HV318	Ig heavy chain V-III region TUR	9.78	12537	68	1	16.4	2	948.9042	1895.7938	1895.0112	0.7826	0	68	EVQLLESGGGLVQPGGSLR
2923	1	P02768	ALBU	Albumin	5.92	71317	1278	42	38.6	1	695.384	694.3767	694.3286	0.0482	0	35	NYAEAK
	1	P02	AL	Albumin	5.	71	12	42	38.6	2	464.	926.	926.	0.0	0	38	YLYEIAR

	768	BU		92	317	78				2781	5416	4861	555			
1	P02768	ALBU	Albumin	5.92	71317	1278	42	38.6	1	940.518	939.5107	939.441	0.0697	0	39	DDNPNLPR
1	P02768	ALBU	Albumin	5.92	71317	1278	42	38.6	2	470.8896	939.7646	939.441	0.3237	0	36	DDNPNLPR
1	P02768	ALBU	Albumin	5.92	71317	1278	42	38.6	2	470.9656	939.9166	939.441	0.4757	0	36	DDNPNLPR
1	P02768	ALBU	Albumin	5.92	71317	1278	42	38.6	1	951.477	950.4697	950.4345	0.0352	0	46	DLGEENFK
1	P02768	ALBU	Albumin	5.92	71317	1278	42	38.6	2	476.3671	950.7196	950.4345	0.2851	0	35	DLGEENFK
1	P02768	ALBU	Albumin	5.92	71317	1278	42	38.6	2	481.0506	960.0866	959.5552	0.5314	0	61	FQNALLVR
1	P02768	ALBU	Albumin	5.92	71317	1278	42	38.6	2	481.2301	960.4456	959.5552	0.8904	0	48	FQNALLVR
1	P02768	ALBU	Albumin	5.92	71317	1278	42	38.6	2	507.3536	1012.6926	1012.5917	0.101	0	82	LVAASQAALGL
1	P02768	ALBU	Albumin	5.92	71317	1278	42	38.6	1	1017.656	1016.6487	1016.5291	0.1196	0	38	SLHTLFGDK
1	P02768	ALBU	Albumin	5.92	71317	1278	42	38.6	2	509.7191	1017.4236	1016.5291	0.8945	0	41	SLHTLFGDK
1	P02768	ALBU	Albumin	5.92	71317	1278	42	38.6	2	538.1077	1074.2008	1073.5353	0.6656	1	46	LDELRDEGK
1	P02768	ALBU	Albumin	5.92	71317	1278	42	38.6	2	565.0142	1128.0138	1127.6914	0.3225	1	55	KQTALVELVK
1	P02768	ALBU	Albumin	5.92	71317	1278	42	38.6	2	571.5677	1141.1208	1140.6866	0.4342	1	68	KLVAASQAALGL
1	P02	AL	Albumin	5.	71	12	42	38.6	2	575.	1149	1148	0.5	0	48	LVNEVTEFAK

	768	BU		92	317	78				5682	.1218	.6077	141				
1	P02768	ALBU	Albumin	5.92	71317	1278	42	38.6	2	613.9312	1225.8478	1225.5979	0.25	1	53	FKDLGEENFK	
1	P02768	ALBU	Albumin	5.92	71317	1278	42	38.6	2	614.3901	1226.7656	1225.5979	1.1678	1	35	FKDLGEENFK	
1	P02768	ALBU	Albumin	5.92	71317	1278	42	38.6	2	656.5787	1311.1428	1310.7347	0.4082	0	53	HPDYSVVLLLR	
1	P02768	ALBU	Albumin	5.92	71317	1278	42	38.6	2	671.8347	1341.6548	1341.6275	0.0274	0	47	AVMDDFAAFVEK	
1	P02768	ALBU	Albumin	5.92	71317	1278	42	38.6	2	672.1227	1342.2308	1341.6275	0.6034	0	81	AVMDDFAAFVEK	
1	P02768	ALBU	Albumin	5.92	71317	1278	42	38.6	2	680.1777	1358.3408	1357.6224	0.7184	0	63	AVMDDFAAFVEK	Oxidation (M)
1	P02768	ALBU	Albumin	5.92	71317	1278	42	38.6	3	490.0165	1467.0277	1466.8358	0.1919	1	35	RHPDYSVVLLLR	
1	P02768	ALBU	Albumin	5.92	71317	1278	42	38.6	2	734.6136	1467.2126	1466.8358	0.3769	1	47	RHPDYSVVLLLR	
1	P02768	ALBU	Albumin	5.92	71317	1278	42	38.6	2	756.7072	1511.3998	1510.8355	0.5643	0	63	VPQVSTPTLVEVSR	
1	P02768	ALBU	Albumin	5.92	71317	1278	42	38.6	2	812.5887	1623.1628	1622.7803	0.3826	0	67	DVFLGMFLYEYAR	
1	P02768	ALBU	Albumin	5.92	71317	1278	42	38.6	2	820.6011	1639.1876	1638.7752	0.4124	0	76	DVFLGMFLYEYAR	Oxidation (M)
1	P02768	ALBU	Albumin	5.92	71317	1278	42	38.6	2	820.7942	1639.5738	1638.9305	0.6434	1	79	KVPQVSTPTLVEVSR	
1	P02768	ALBU	Albumin	5.92	71317	1278	42	38.6	3	547.6259	1639.8559	1638.9305	0.9254	1	66	KVPQVSTPTLVEVSR	
1	P02	AL	Albumin	5.	71	12	42	38.6	2	828.	1655	1656	-1.	0	55	QNCELFEQLGEYK	

	768	BU		92	317	78				7382	.4618	.7453	2835				
1	P02768	ALBU	Albumin	5.92	71317	1278	42	38.6	3	581.9805	1742.9197	1741.8868	1.0329	0	50	HPYFYAPELLFFAK	
1	P02768	ALBU	Albumin	5.92	71317	1278	42	38.6	2	872.8242	1743.6338	1741.8868	1.7471	0	39	HPYFYAPELLFFAK	
1	P02768	ALBU	Albumin	5.92	71317	1278	42	38.6	3	633.9505	1898.8297	1897.9879	0.8418	1	47	RHPYFYAPELLFFAK	
1	P02768	ALBU	Albumin	5.92	71317	1278	42	38.6	3	682.8782	2045.6128	2044.0881	1.5247	0	37	VFDEFKPLVEEPQNLIK	
1	P02768	ALBU	Albumin	5.92	71317	1278	42	38.6	3	682.9965	2045.9677	2044.0881	1.8796	0	47	VFDEFKPLVEEPQNLIK	
1	P02768	ALBU	Albumin	5.92	71317	1278	42	38.6	2	1333.195	2664.3754	2665.2516	-0.8762	0	45	LVRPEVDVMCTAFHDNEETFLK	Oxidation (M)
1	P02768	ALBU	Albumin	5.92	71317	1278	42	38.6	3	889.4428	2665.3066	2665.2516	0.0549	0	39	LVRPEVDVMCTAFHDNEETFLK	Oxidation (M)
1	P02768	ALBU	Albumin	5.92	71317	1278	42	38.6	3	889.5695	2665.6867	2665.2516	0.435	0	43	LVRPEVDVMCTAFHDNEETFLK	Oxidation (M)
1	P02768	ALBU	Albumin	5.92	71317	1278	42	38.6	3	991.7798	2972.3176	2973.3372	-1.0196	0	35	SHCIAEVENDEMPADLPSLAADFVESK	
1	P02768	ALBU	Albumin	5.92	71317	1278	42	38.6	3	997.0289	2988.0649	2989.3321	-1.2672	0	35	SHCIAEVENDEMPADLPSLAADFVESK	Oxidation (M)
1	P02768	ALBU	Albumin	5.92	71317	1278	42	38.6	3	997.1035	2988.2887	2989.3321	-1.0434	0	37	SHCIAEVENDEMPADLPSLAADFVESK	Oxidation (M)
1	P02768	ALBU	Albumin	5.92	71317	1278	42	38.6	3	998.0705	2991.1897	2989.3321	1.8576	0	46	SHCIAEVENDEMPADLPSLAADFVESK	Oxidation (M)
2	P04264	K2C1	Keratin, type II cytoskeletal 1	8.15	66170	929	14	31.4	2	487.4851	972.9556	972.524	0.4317	0	48	IEISELNR	

2	P04264	K2C1	Keratin, type II cytoskeletal 1	8.15	66170	929	14	31.4	2	517.6222	1033.2298	1032.5087	0.72111	0	37	TLLEGEESR
2	P04264	K2C1	Keratin, type II cytoskeletal 1	8.15	66170	929	14	31.4	2	571.6971	1141.3796	1140.5121	0.86775	0	44	DYQELMNTK
2	P04264	K2C1	Keratin, type II cytoskeletal 1	8.15	66170	929	14	31.4	2	590.6307	1179.2468	1178.5931	0.65537	0	75	YEELQITAGR
2	P04264	K2C1	Keratin, type II cytoskeletal 1	8.15	66170	929	14	31.4	2	633.7317	1265.4488	1264.6299	0.81899	0	58	TNAENEFVTIK
2	P04264	K2C1	Keratin, type II cytoskeletal 1	8.15	66170	929	14	31.4	2	651.0922	1300.1698	1299.5224	0.64775	0	72	NMQDMVEDYR
2	P04264	K2C1	Keratin, type II cytoskeletal 1	8.15	66170	929	14	31.4	2	652.3957	1302.7768	1301.7078	1.069	0	96	SLDLDSIIAEVK
2	P04264	K2C1	Keratin, type II cytoskeletal 1	8.15	66170	929	14	31.4	2	671.7051	1341.3956	1339.6619	1.7337	1	57	SKAEAESLYQSK
2	P04264	K2C1	Keratin, type II cytoskeletal 1	8.15	66170	929	14	31.4	2	679.7402	1357.4658	1356.6885	0.77774	0	84	LNDLEDALQQAK
2	P04264	K2C1	Keratin, type II cytoskeletal 1	8.15	66170	929	14	31.4	2	692.7776	1383.5406	1382.683	0.8576	0	63	SLNNQFASFIDK
2	P04264	K2C1	Keratin, type II cytoskeletal 1	8.15	66170	929	14	31.4	2	738.6841	1475.3536	1474.778	0.5757	0	78	FLEQQNQVLQTK
2	P04264	K2C1	Keratin, type II	8.1	6617	929	14	31.4	2	859.145	1716.275	1715.843	0.431	0	69	QISNLQQSISDAEQR

			cytoskeletal 1	5	0					1	6	8	8				
2	P04264	K2C1	Keratin, type II cytoskeletal 1	8.15	66170	929	14	31.4	2	1193.001	2383.9874	2382.9447	1.0428	0	80	GGGGGGYGSGGSSYGSGGGSYGSGGGGGGGR	
2	P04264	K2C1	Keratin, type II cytoskeletal 1	8.15	66170	929	14	31.4	3	1105.258	3312.7522	3311.3009	1.4513	0	85	GSYGSGGSSYGSGGGSYGSGGGGGGHGSYGSGSSSGGYR	
3	P35527	K1C9	Keratin, type I cytoskeletal 9	5.14	62255	829	15	32.1	2	531.0662	1060.1178	1059.556	0.5618	0	46	TLLDIDNTR	
3	P35527	K1C9	Keratin, type I cytoskeletal 9	5.14	62255	829	15	32.1	2	533.6327	1065.2508	1064.492	0.7588	0	44	STMQELNSR	
3	P35527	K1C9	Keratin, type I cytoskeletal 9	5.14	62255	829	15	32.1	2	561.6036	1121.1926	1120.5764	0.6162	0	38	QEYEQLIAK	
3	P35527	K1C9	Keratin, type I cytoskeletal 9	5.14	62255	829	15	32.1	2	579.3607	1156.7068	1156.5836	0.1232	0	60	QGVDADINGLR	
3	P35527	K1C9	Keratin, type I cytoskeletal 9	5.14	62255	829	15	32.1	2	603.9877	1205.9608	1205.5962	0.3647	0	48	QVLDNLTMEK	Oxidation (M)
3	P35527	K1C9	Keratin, type I cytoskeletal 9	5.14	62255	829	15	32.1	2	617.1757	1232.3368	1231.5906	0.7463	0	63	SGGGGGGGLGSGGSIR	
3	P35527	K1C9	Keratin, type I cytoskeletal 9	5.14	62255	829	15	32.1	2	618.6741	1235.3336	1234.5215	0.8122	0	74	FSSSSGYGGGSSR	
3	P35527	K1C9	Keratin, type I cytoskeletal 9	5.14	62255	829	15	32.1	2	794.2642	1586.5138	1585.7583	0.7555	0	89	VQALEEANNDLENK	

3	P35527	K1C9	Keratin, type I cytoskeletal 9	5.14	62255	829	15	32.1	2	896.6312	1791.2478	1790.7205	0.5274	0	99	GGSGGSYGGGGSGGGYGGGSGSR	
3	P35527	K1C9	Keratin, type I cytoskeletal 9	5.14	62255	829	15	32.1	3	613.6082	1837.8028	1836.9581	0.8447	0	54	HGVQELEIELQSQLSK	
3	P35527	K1C9	Keratin, type I cytoskeletal 9	5.14	62255	829	15	32.1	2	1094.427	2186.8394	2186.0122	0.8272	0	63	SDLEMQYETLQEELMALK	Oxidation (M)
3	P35527	K1C9	Keratin, type I cytoskeletal 9	5.14	62255	829	15	32.1	2	1102.396	2202.7774	2202.0072	0.7703	0	55	SDLEMQYETLQEELMALK	2 Oxidation (M)
3	P35527	K1C9	Keratin, type I cytoskeletal 9	5.14	62255	829	15	32.1	2	1255.569	2509.1234	2509.1245	-0.001	0	35	EIETYHNLLEGGQEDFESSGAGK	
3	P35527	K1C9	Keratin, type I cytoskeletal 9	5.14	62255	829	15	32.1	3	837.6815	2510.0227	2509.1245	0.8982	0	67	EIETYHNLLEGGQEDFESSGAGK	
3	P35527	K1C9	Keratin, type I cytoskeletal 9	5.14	62255	829	15	32.1	3	1089.359	3265.0552	3263.5066	1.5486	0	94	DIENQYETQITQIEHEVSSSGQEVQSSAK	
4	P13645	K1C10	Keratin, type I cytoskeletal 10	5.09	59046	303	4	10.1	2	546.2142	1090.4138	1089.5237	0.8902	0	41	VTMQNLNDR	
4	P13645	K1C10	Keratin, type I cytoskeletal 10	5.09	59046	303	4	10.1	2	632.1476	1262.2806	1261.5899	0.6908	0	69	SLLEGEGSSGGGGR	
4	P13645	K1C10	Keratin, type I cytoskeletal 10	5.09	59046	303	4	10.1	2	854.5461	1707.0776	1706.7649	0.3127	0	115	GSLGGGFSSGGFSGGSFSR	
4	P13645	K1C1	Keratin, type I	5.0	5904	303	4	10.1	2	999.409	1996.803	1995.963	0.840	0	78	ELTTEIDNNIEQISSYK	

			0	cytoskeletal 10	9	6					2	8	6	2				
	5	P02790	HEMO	Hemopexin	6.555	52385	151	4	8.4	2	571.6042	1141.1938	1140.5815	0.6123	0	59	GGYTLVSGYPK	
	5	P02790	HEMO	Hemopexin	6.555	52385	151	4	8.4	2	742.9742	1483.9338	1483.6806	0.2533	0	50	EWFWDLATGTMK	
	5	P02790	HEMO	Hemopexin	6.555	52385	151	4	8.4	2	751.2012	1500.3878	1499.6755	0.7123	0	52	EWFWDLATGTMK	Oxidation (M)
	5	P02790	HEMO	Hemopexin	6.555	52385	151	4	8.4	3	613.6841	1838.0305	1836.8795	1.151	0	45	SGAQATWTELPWPHEK	
2936	1	P02768	ALBU	Albumin	5.92	71317	1045	23	33.7	1	695.461	694.4537	694.3286	0.1252	0	40	NYAEAK	
	1	P02768	ALBU	Albumin	5.92	71317	1045	23	33.7	1	951.659	950.6517	950.4345	0.2172	0	35	DLGEENFK	
	1	P02768	ALBU	Albumin	5.92	71317	1045	23	33.7	2	481.1326	960.2506	959.5552	0.6954	0	45	FQNALLVR	
	1	P02768	ALBU	Albumin	5.92	71317	1045	23	33.7	2	501.2791	1000.5436	999.5964	0.9472	0	59	QTALVELVK	
	1	P02768	ALBU	Albumin	5.92	71317	1045	23	33.7	2	507.6501	1013.2856	1012.5917	0.694	0	79	LVAASQAALGL	
	1	P02768	ALBU	Albumin	5.92	71317	1045	23	33.7	2	509.4091	1016.8036	1016.5291	0.2745	0	38	SLHTLFGDK	
	1	P02768	ALBU	Albumin	5.92	71317	1045	23	33.7	2	538.5557	1075.0968	1073.5353	1.5616	1	47	LDELRDEGK	
	1	P02768	ALBU	Albumin	5.92	71317	1045	23	33.7	2	575.7756	1149.5366	1148.6077	0.9289	0	52	LVNEVTEFAK	
	1	P02768	ALBU	Albumin	5.92	71317	1045	23	33.7	2	614.2776	1226.5406	1225.5979	0.9428	1	45	FKDLGEENFK	
	1	P02	AL	Albumin	5.	71	10	23	33.7	2	656.	1311	1310	0.8	0	47	HPDYSVVLLLR	

	768	BU		92	317	45				8102	.6058	.7347	712				
1	P02768	ALBU	Albumin	5.92	71317	1045	23	33.7	2	672.1652	1342.3158	1341.6275	0.6884	0	73	AVMDDFAAFVEK	
1	P02768	ALBU	Albumin	5.92	71317	1045	23	33.7	2	680.1816	1358.3486	1357.6224	0.7262	0	59	AVMDDFAAFVEK	Oxidation (M)
1	P02768	ALBU	Albumin	5.92	71317	1045	23	33.7	3	490.2382	1467.6928	1466.8358	0.857	1	63	RHPDYSVVLLLR	
1	P02768	ALBU	Albumin	5.92	71317	1045	23	33.7	2	735.0281	1468.0416	1466.8358	1.2059	1	48	RHPDYSVVLLLR	
1	P02768	ALBU	Albumin	5.92	71317	1045	23	33.7	2	756.6036	1511.1926	1510.8355	0.3571	0	70	VPQVSTPTLVEVSR	
1	P02768	ALBU	Albumin	5.92	71317	1045	23	33.7	2	820.4177	1638.8208	1638.7752	0.0456	0	74	DVFLGMFLYEYAR	Oxidation (M)
1	P02768	ALBU	Albumin	5.92	71317	1045	23	33.7	3	547.4761	1639.4065	1638.9305	0.476	1	59	KVPQVSTPTLVEVSR	
1	P02768	ALBU	Albumin	5.92	71317	1045	23	33.7	2	820.8326	1639.6506	1638.9305	0.7202	1	70	KVPQVSTPTLVEVSR	
1	P02768	ALBU	Albumin	5.92	71317	1045	23	33.7	3	581.9858	1742.9356	1741.8868	1.0488	0	43	HPYFYAPELLFFAK	
1	P02768	ALBU	Albumin	5.92	71317	1045	23	33.7	3	634.0262	1899.0568	1897.9879	1.0689	1	44	RHPYFYAPELLFFAK	
1	P02768	ALBU	Albumin	5.92	71317	1045	23	33.7	3	682.9329	2045.7769	2044.0881	1.6888	0	46	VFDEFKPLVEEPQNLIK	
1	P02768	ALBU	Albumin	5.92	71317	1045	23	33.7	3	890.0045	2666.9917	2665.2516	1.74	0	53	LVRPEVDVMCTAFHDNEETFLK	Oxidation (M)
1	P02768	ALBU	Albumin	5.92	71317	1045	23	33.7	3	997.8008	2990.3806	2989.3321	1.0485	0	51	SHCIAEVENDEMPADLPSLAADFVESK	Oxidation (M)
2	P05	HE	Heparin	6.	57	45	10	21.4	2	450.	899.	898.	0.5	0	43	GPLDQLEK	

	546	P2	cofactor 2	41	205	8				5376	0606	476	847				
2	P05546	HEP2	Heparin cofactor 2	6.41	57205	458	10	21.4	2	466.1156	930.2166	929.4759	0.7407	0	45	FAFNLYR	
2	P05546	HEP2	Heparin cofactor 2	6.41	57205	458	10	21.4	2	515.0627	1028.1108	1027.5662	0.5447	0	56	TLEAQLTPR	
2	P05546	HEP2	Heparin cofactor 2	6.41	57205	458	10	21.4	2	540.5527	1079.0908	1078.5658	0.525	0	45	NYNLVESLK	
2	P05546	HEP2	Heparin cofactor 2	6.41	57205	458	10	21.4	2	540.6967	1079.3788	1078.5659	0.813	0	45	SVNDLYIQK	
2	P05546	HEP2	Heparin cofactor 2	6.41	57205	458	10	21.4	2	561.1246	1120.2346	1119.6328	0.6018	0	37	QFPILLDFK	
2	P05546	HEP2	Heparin cofactor 2	6.41	57205	458	10	21.4	2	704.5582	1407.1018	1405.7354	1.3665	0	45	YEITTIHNLFR	
2	P05546	HEP2	Heparin cofactor 2	6.41	57205	458	10	21.4	3	510.6888	1529.0446	1527.7041	1.3405	0	44	FPVEMTHNHNFR	
2	P05546	HEP2	Heparin cofactor 2	6.41	57205	458	10	21.4	3	652.0395	1953.0967	1952.0309	1.0658	0	61	FTVDRPFLFLIYEHR	
2	P05546	HEP2	Heparin cofactor 2	6.41	57205	458	10	21.4	2	987.2172	1972.4198	1971.8922	0.5276	0	47	GGETAQSADPQWEQLNNK	
3	P02790	HEMO	Hemopexin	6.55	52385	260	7	12.6	2	565.7496	1129.4846	1128.6444	0.8403	1	34	RLWWLDLK	
3	P02790	HEMO	Hemopexin	6.55	52385	260	7	12.6	2	571.6672	1141.3198	1140.5815	0.7383	0	56	GGYTLVSGYPK	
3	P02790	HEMO	Hemopexin	6.55	52385	260	7	12.6	2	611.1371	1220.2596	1219.5986	0.6611	0	44	NFPSPVDAAFR	
3	P02790	HEMO	Hemopexin	6.55	52385	260	7	12.6	2	743.0042	1483.9938	1483.6806	0.3133	0	64	EWFWDLATGTMK	
3	P02	HE	Hemope	6.	52	26	7	12.6	2	750.	1499	1499	0.1	0	65	EWFWDLATGTMK	Oxida

		790	MO	xin	55	385	0				9232	.8318	.6755	563				tion (M)
	3	P02790	HEMO	Hemopexin	6.55	52385	260	7	12.6	3	613.5851	1837.7335	1836.8795	0.854	0	56	SGAQATWTELPWPHEK	
	3	P02790	HEMO	Hemopexin	6.55	52385	260	7	12.6	2	919.8752	1837.7358	1836.8795	0.8564	0	66	SGAQATWTELPWPHEK	
2937	1	P02768	ALBU	Albumin	5.92	71317	1267	34	38.6	1	789.59	788.5827	788.4644	0.1184	0	39	LVTDLTK	
	1	P02768	ALBU	Albumin	5.92	71317	1267	34	38.6	1	880.464	879.4567	879.4338	0.0229	0	34	AEFAEVSK	
	1	P02768	ALBU	Albumin	5.92	71317	1267	34	38.6	2	464.5486	927.0826	926.4861	0.5965	0	38	YLYEIAR	
	1	P02768	ALBU	Albumin	5.92	71317	1267	34	38.6	2	476.4636	950.9126	950.4345	0.4781	0	36	DLGEENFK	
	1	P02768	ALBU	Albumin	5.92	71317	1267	34	38.6	2	481.1636	960.3126	959.5552	0.7574	0	48	FQNALLVR	
	1	P02768	ALBU	Albumin	5.92	71317	1267	34	38.6	2	501.0546	1000.0946	999.5964	0.4982	0	71	QTALVELVK	
	1	P02768	ALBU	Albumin	5.92	71317	1267	34	38.6	2	507.5561	1013.0976	1012.5917	0.506	0	95	LVAASQAALGL	
	1	P02768	ALBU	Albumin	5.92	71317	1267	34	38.6	1	1017.462	1016.4547	1016.5291	-0.0744	0	45	SLHTLFGDK	
	1	P02768	ALBU	Albumin	5.92	71317	1267	34	38.6	2	509.6056	1017.1966	1016.5291	0.6675	0	41	SLHTLFGDK	
	1	P02768	ALBU	Albumin	5.92	71317	1267	34	38.6	2	528.5652	1055.1158	1054.5811	0.5348	1	34	KYLYEIAR	
	1	P02768	ALBU	Albumin	5.92	71317	1267	34	38.6	2	537.9161	1073.8176	1073.5353	0.2824	1	45	LDELRDEGK	
	1	P02	AL	Albumin	5.	71	12	34	38.6	2	565.	1128	1127	0.6	1	57	KQTALVELVK	

	768	BU		92	317	67				1822	.3498	.6914	585				
1	P02768	ALBU	Albumin	5.92	71317	1267	34	38.6	2	575.6277	1149.2408	1148.6077	0.6331	0	48	LVNEVTEFAK	
1	P02768	ALBU	Albumin	5.92	71317	1267	34	38.6	2	614.1871	1226.3596	1225.5979	0.7618	1	51	FKDLGEENFK	
1	P02768	ALBU	Albumin	5.92	71317	1267	34	38.6	2	656.8412	1311.6678	1310.7347	0.9332	0	50	HPDYSVVLLLR	
1	P02768	ALBU	Albumin	5.92	71317	1267	34	38.6	1	1342.781	1341.7737	1341.6275	0.1462	0	39	AVMDDFAAFVEK	
1	P02768	ALBU	Albumin	5.92	71317	1267	34	38.6	2	672.0132	1342.0118	1341.6275	0.3844	0	60	AVMDDFAAFVEK	
1	P02768	ALBU	Albumin	5.92	71317	1267	34	38.6	2	679.9316	1357.8486	1357.6224	0.2262	0	69	AVMDDFAAFVEK	Oxidation (M)
1	P02768	ALBU	Albumin	5.92	71317	1267	34	38.6	2	734.8641	1467.7136	1466.8358	0.8779	1	37	RHPDYSVVLLLR	
1	P02768	ALBU	Albumin	5.92	71317	1267	34	38.6	3	490.3552	1468.0438	1466.8358	1.208	1	63	RHPDYSVVLLLR	
1	P02768	ALBU	Albumin	5.92	71317	1267	34	38.6	2	756.5856	1511.1566	1510.8355	0.3211	0	73	VPQVSTPTLVEVSR	
1	P02768	ALBU	Albumin	5.92	71317	1267	34	38.6	3	504.9815	1511.9227	1510.8355	1.0871	0	43	VPQVSTPTLVEVSR	
1	P02768	ALBU	Albumin	5.92	71317	1267	34	38.6	2	813.2012	1624.3878	1622.7803	1.6076	0	69	DVFLGMFLYEYAR	
1	P02768	ALBU	Albumin	5.92	71317	1267	34	38.6	2	820.6896	1639.3646	1638.7752	0.5894	0	76	DVFLGMFLYEYAR	Oxidation (M)
1	P02768	ALBU	Albumin	5.92	71317	1267	34	38.6	3	547.5541	1639.6405	1638.9305	0.71	1	68	KVPQVSTPTLVEVSR	
1	P02	AL	Albumin	5.	71	12	34	38.6	3	547.	1640	1638	1.1	1	62	KVPQVSTPTLVEVSR	

	768	BU		92	317	67				6962	.0668	.9305	363				
1	P02768	ALBU	Albumin	5.92	71317	1267	34	38.6	2	821.4186	1640.8226	1638.9305	1.8922	1	75	KVPQVSTPTLVEVSR	
1	P02768	ALBU	Albumin	5.92	71317	1267	34	38.6	2	828.3862	1654.7578	1656.7453	-1.9875	0	41	QNCELFEQLGEYK	
1	P02768	ALBU	Albumin	5.92	71317	1267	34	38.6	2	872.1917	1742.3688	1741.8868	0.4821	0	50	HPYFYAPELLFFAK	
1	P02768	ALBU	Albumin	5.92	71317	1267	34	38.6	3	581.9125	1742.7157	1741.8868	0.8289	0	62	HPYFYAPELLFFAK	
1	P02768	ALBU	Albumin	5.92	71317	1267	34	38.6	3	682.8925	2045.6557	2044.0881	1.5676	0	37	VFDEFKPLVEEPQNLIK	
1	P02768	ALBU	Albumin	5.92	71317	1267	34	38.6	3	888.8245	2663.4517	2665.2516	-1.8	0	48	LVRPEVDVMCTAFHDNEETFLK	Oxidation (M)
1	P02768	ALBU	Albumin	5.92	71317	1267	34	38.6	2	1332.881	2663.7474	2665.2516	-1.5042	0	48	LVRPEVDVMCTAFHDNEETFLK	Oxidation (M)
1	P02768	ALBU	Albumin	5.92	71317	1267	34	38.6	3	996.8378	2987.4916	2989.3321	-1.8405	0	46	SHCIAEVENDEMPADLPSLAADFVESK	Oxidation (M)
2	P05546	HEP2	Heparin cofactor 2	6.41	57205	540	11	25.7	2	450.5511	899.0876	898.476	0.6117	0	52	GPLDQLEK	
2	P05546	HEP2	Heparin cofactor 2	6.41	57205	540	11	25.7	2	466.1896	930.3646	929.4759	0.8887	0	46	FAFNLYR	
2	P05546	HEP2	Heparin cofactor 2	6.41	57205	540	11	25.7	2	515.0222	1028.0298	1027.5662	0.4637	0	53	TLEAQLTPR	
2	P05546	HEP2	Heparin cofactor 2	6.41	57205	540	11	25.7	2	540.5486	1079.0826	1078.5659	0.5168	0	56	SVNDLYIQK	
2	P05546	HEP2	Heparin cofactor 2	6.41	57205	540	11	25.7	2	561.4432	1120.8718	1119.6328	1.239	0	39	QFPILLDFK	
2	P05	HE	Heparin	6.	57	54	11	25.7	2	704.	1406	1405	0.5	0	49	YEITTIHNLFR	

	546	P2	cofactor 2	41	205	0				1692	.3238	.7354	885				
2	P05546	HEP2	Heparin cofactor 2	6.41	57205	540	11	25.7	3	510.5385	1528.5937	1527.7041	0.8896	0	47	FPVEMTHNHNFR	
2	P05546	HEP2	Heparin cofactor 2	6.41	57205	540	11	25.7	3	652.2319	1953.6739	1952.0309	1.643	0	74	FTVDRPFLFLIYEHR	
2	P05546	HEP2	Heparin cofactor 2	6.41	57205	540	11	25.7	2	987.2682	1972.5218	1971.8922	0.6296	0	72	GGETAQSADPQWEQLNNK	
2	P05546	HEP2	Heparin cofactor 2	6.41	57205	540	11	25.7	3	1068.583	3202.7272	3202.5929	0.1343	0	48	HQGTITVNEEGTQATTVTTVGFMPLSTQVR	
2	P05546	HEP2	Heparin cofactor 2	6.41	57205	540	11	25.7	3	1074.477	3220.4092	3218.5878	1.8214	0	58	HQGTITVNEEGTQATTVTTVGFMPLSTQVR	Oxidation (M)
3	P02790	HEMO	Hemopexin	6.55	52385	419	10	18.0	2	565.7557	1129.4968	1128.6444	0.8525	1	44	RLWWLDLK	
3	P02790	HEMO	Hemopexin	6.55	52385	419	10	18.0	1	1141.541	1140.5337	1140.5815	-0.0478	0	43	GGYTLVSGYPK	
3	P02790	HEMO	Hemopexin	6.55	52385	419	10	18.0	2	571.4362	1140.8578	1141.6244	-0.7665	0	46	QGHNSVFLIK	
3	P02790	HEMO	Hemopexin	6.55	52385	419	10	18.0	2	571.6376	1141.2606	1140.5815	0.6791	0	64	GGYTLVSGYPK	
3	P02790	HEMO	Hemopexin	6.55	52385	419	10	18.0	2	611.1072	1220.1998	1219.5986	0.6013	0	37	NFPSPVDAAFR	
3	P02790	HEMO	Hemopexin	6.55	52385	419	10	18.0	2	743.1597	1484.3048	1483.6806	0.6243	0	64	EWFWDLATGTMK	
3	P02790	HEMO	Hemopexin	6.55	52385	419	10	18.0	2	750.9847	1499.9548	1499.6755	0.2793	0	76	EWFWDLATGTMK	Oxidation (M)
3	P02790	HEMO	Hemopexin	6.55	52385	419	10	18.0	2	886.7676	1771.5206	1770.992	0.5286	0	96	LYLVQGTQVYVFLTK	
3	P02	HE	Hemope	6.	52	41	10	18.0	3	613.	1837	1836	0.5	0	50	SGAQATWTELPWPHEK	

		790	MO	xin	55	385	9				4758	.4056	.8795	261				
	3	P02790	HEMO	Hemopexin	6.55	52385	419	10	18.0	2	919.8477	1837.6808	1836.8795	0.8014	0	66	SGAQATWTELPWPHEK	
2938	1	P02768	ALBU	Albumin	5.92	71317	259	6	10.8	2	575.6172	1149.2198	1148.6077	0.6121	0	47	LVNEVTEFAK	
	1	P02768	ALBU	Albumin	5.92	71317	259	6	10.8	2	672.2057	1342.3968	1341.6275	0.7694	0	68	AVMDDFAAFVEK	
	1	P02768	ALBU	Albumin	5.92	71317	259	6	10.8	2	680.3237	1358.6328	1357.6224	1.0104	0	54	AVMDDFAAFVEK	Oxidation (M)
	1	P02768	ALBU	Albumin	5.92	71317	259	6	10.8	3	490.4509	1468.3309	1466.8358	1.4951	1	63	RHPDYSVVLLLR	
	1	P02768	ALBU	Albumin	5.92	71317	259	6	10.8	3	547.5778	1639.7116	1638.9305	0.7811	1	51	KVPQVSTPTLVEVSR	
	1	P02768	ALBU	Albumin	5.92	71317	259	6	10.8	2	1023.353	2044.6914	2044.0881	0.6034	0	36	VFDEFKPLVEEPQNLIK	
	2	P00739	HPTR	Haptoglobin-related protein	6.42	39496	161	4	4.3	2	656.6296	1311.2446	1310.599	0.6456	0	69	TEGDGVYTLNDK	
	2	P00739	HPTR	Haptoglobin-related protein	6.42	39496	161	4	4.3	2	720.7126	1439.4106	1438.694	0.7167	1	51	TEGDGVYTLNDKK	
	2	P00739	HPTR	Haptoglobin-related protein	6.42	39496	161	4	4.3	2	720.8027	1439.5908	1438.694	0.8969	1	35	TEGDGVYTLNDKK	
2962	1	P04004	VTNC	Vitronectin	5.55	55069	195	7	9.0	2	711.8542	1421.6938	1421.6463	0.0475	0	65	FEDGVLDPDYPR	
	1	P04004	VTNC	Vitronectin	5.55	55069	195	7	9.0	2	712.0071	1421.9996	1421.6463	0.3533	0	62	FEDGVLDPDYPR	
	1	P04	VT	Vitronect	5.	55	19	7	9.0	2	824.	1646	1645	0.5	0	82	DVWGIEGPIDAAFTR	

		004	NC	in	55	069	5				1736	.3326	.81	226				
	1	P04004	VTNC	Vitronectin	5.55	55069	195	7	9.0	3	556.5068	1666.4986	1665.7682	0.7304	0	39	DWHGVPGQVDAAMAGR	
	1	P04004	VTNC	Vitronectin	5.55	55069	195	7	9.0	2	834.8157	1667.6168	1665.7682	1.8487	0	51	DWHGVPGQVDAAMAGR	
	1	P04004	VTNC	Vitronectin	5.55	55069	195	7	9.0	3	561.4142	1681.2208	1681.7631	-0.5423	0	34	DWHGVPGQVDAAMAGR	Oxidation (M)
	1	P04004	VTNC	Vitronectin	5.55	55069	195	7	9.0	3	561.9445	1682.8117	1681.7631	1.0486	0	52	DWHGVPGQVDAAMAGR	Oxidation (M)
2963	1	P04004	VTNC	Vitronectin	5.55	55069	172	5	9.0	2	712.2212	1422.4278	1421.6463	0.7815	0	48	FEDGVLDPDYPR	
	1	P04004	VTNC	Vitronectin	5.55	55069	172	5	9.0	2	824.8857	1647.7568	1645.81	1.9468	0	67	DVWGIEGPIDAAFTR	
	1	P04004	VTNC	Vitronectin	5.55	55069	172	5	9.0	2	833.9897	1665.9648	1665.7682	0.1967	0	60	DWHGVPGQVDAAMAGR	
	1	P04004	VTNC	Vitronectin	5.55	55069	172	5	9.0	3	556.4212	1666.2418	1665.7682	0.4736	0	55	DWHGVPGQVDAAMAGR	
	1	P04004	VTNC	Vitronectin	5.55	55069	172	5	9.0	3	561.6602	1681.9588	1681.7631	0.1957	0	59	DWHGVPGQVDAAMAGR	Oxidation (M)

Supplemental Table 7 Reported concentration of identified proteins and references

protein name	normal concentration μg/ml	References
adiponectin	2-17	[1]
albumin	35000-52000	[2]
alpha-1-antitrypsin	900-2000	[3]
alpha-1B-glycoprotein	150-300	[2]
alpha-2-macroglobulin	1300-3000	[3]
apolipoprotein A-I	1000-2000	[2]
apolipoprotein A-II	190-300	[4]
apolipoprotein A-IV	110-220	[5]
apolipoprotein D	60-90	[6]
apolipoprotein E	30-60	[7]
carboxypeptidase N	30	[8]
ceruloplasmin	190-370	[9]
clusterin	250-420	[10]
coagulation factor X	10	[11]
complement C3	900-1800	[2]
complement C4-A	25-90	[3]
fibrinogen beta chain	520-1420	[3]
fibrinogen gamma chain	490-1340	[3]
ficolin-2	1-12	[12]
ficolin-3	3-54	[13]
haptoglobin	200-2000	[14]
haptoglobin-related protein	32-41	[14]
inter-alpha-trypsin inhibitor (heavy chain H3)	100-200	[3]
paraoxonase/arylesterase 1	58-61	[15]
prothrombin	100	[3]
serotransferrin	2000-3600	[2]
transthyretin	200-400	[2]
vitronectin	240-530	[10]
zinc-alpha-2-glycoprotein	60-80	[16]

References

[1] Arita, Y., Kihara, S., Ouchi, N., Takahashi, M., *et al.*, Paradoxical decrease of an adipose-specific protein, adiponectin, in obesity. *Biochemical and Biophysical Research Communications* 1999, *257*, 79-83.

[2] Hortin, G. L., The MALDI-TOF mass spectrometric view of the plasma proteome and peptidome. *Clinical Chemistry* 2006, *52*, 1223-1237.

[3] Hortin, G. L., Sviridov, D., Anderson, N. L., High-abundance polypeptides of the human plasma proteome comprising the top 4 logs of polypeptide abundance. *Clinical Chemistry* 2008, *54*, 1608-1616.

[4] Luo, J., Liu, B., [ELISA for measurement of human serum apolipoprotein A II]. *Hua Xi Yi Ke Da Xue Xue Bao* 1994, *25*, 229-232.

[5] Kondo, K., Allan, C., Fidge, N., QUANTITATION OF APOLIPOPROTEIN-A-IV IN HUMAN-PLASMA USING A COMPETITIVE ENZYME-LINKED IMMUNOSORBENT-ASSAY. *Journal of Lipid Research* 1989, *30*, 939-944.
[6] Cheung, M. C., Albers, J. J., CHARACTERIZATION OF LIPOPROTEIN PARTICLES ISOLATED BY IMMUNOAFFINITY CHROMATOGRAPHY - PARTICLES CONTAINING A-I AND A-II AND PARTICLES CONTAINING A-I BUT NO A-II. *Journal of Biological Chemistry* 1984, *259*, 2201-2209.
[7] Schiele, F., De Bacquer, D., Vincent-Viry, M., Beisiegel, U., *et al.*, Apolipoprotein E serum concentration and polymorphism in six European countries: the ApoEurope Project. *Atherosclerosis* 2000, *152*, 475-488.
[8] Matthews, K. W., Mueller-Ortiz, S. L., Wetsel, R. A., Carboxypeptidase N: a pleiotropic regulator of inflammation. *Molecular Immunology* 2004, *40*, 785-793.
[9] Kim, C. H., Park, J. Y., Kim, J. Y., Choi, C. S., *et al.*, Elevated serum ceruloplasmin levels in subjects with metabolic syndrome: A population-based study. *Metabolism-Clinical and Experimental* 2002, *51*, 838-842.
[10] Hogasen, K., Mollnes, T. E., Tschopp, J., Harboe, M., QUANTITATION OF VITRONECTIN AND CLUSTERIN - PITFALLS AND SOLUTIONS IN ENZYME IMMUNOASSAYS FOR ADHESIVE PROTEINS. *Journal of Immunological Methods* 1993, *160*, 107-115.
[11] Kalafatis, M., Egan, J. O., VantVeer, C., Cawthern, K. M., Mann, K. G., The regulation of clotting factors. *Critical Reviews in Eukaryotic Gene Expression* 1997, *7*, 241-280.
[12] Munthe-Fog, L., Hummelshoj, T., Hansen, B. E., Koch, C., *et al.*, The impact of FCN2 polymorphisms and haplotypes on the ficolin-2 serum levels. *Scandinavian Journal of Immunology* 2007, *65*, 383-392.
[13] Munthe-Fog, L., Hummelshoj, T., Ma, Y. J., Hansen, B. E., *et al.*, Characterization of a polymorphism in the coding sequence of FCN3 resulting in a Ficolin-3 (Hakata antigen) deficiency state. *Molecular Immunology* 2008, *45*, 2660-2666.
[14] Kujiraoka, T., Oka, T., Ishihara, M., Egashira, T., *et al.*, A sandwich enzyme-linked immunosorbent assay for human serum paraoxonase concentration. *Journal of Lipid Research* 2000, *41*, 1358-1363.
[15] Kujiraoka, T., Oka, T., Ishihara, M., Egashira, T., *et al.*, A sandwich enzyme-linked immunosorbent assay for human serum paraoxonase concentration. *Journal of Lipid Research* 2000, *41*, 1358-1363.
[16] Yilmaz, Y., Yonal, O., Eren, F., Kurt, R., *et al.*, Serum zinc-α2-glycoprotein concentrations in patients with non-alcoholic fatty liver disease. *Clin Chem Lab Med* 2011, *49*, 93-97.

Supplementary figure 1

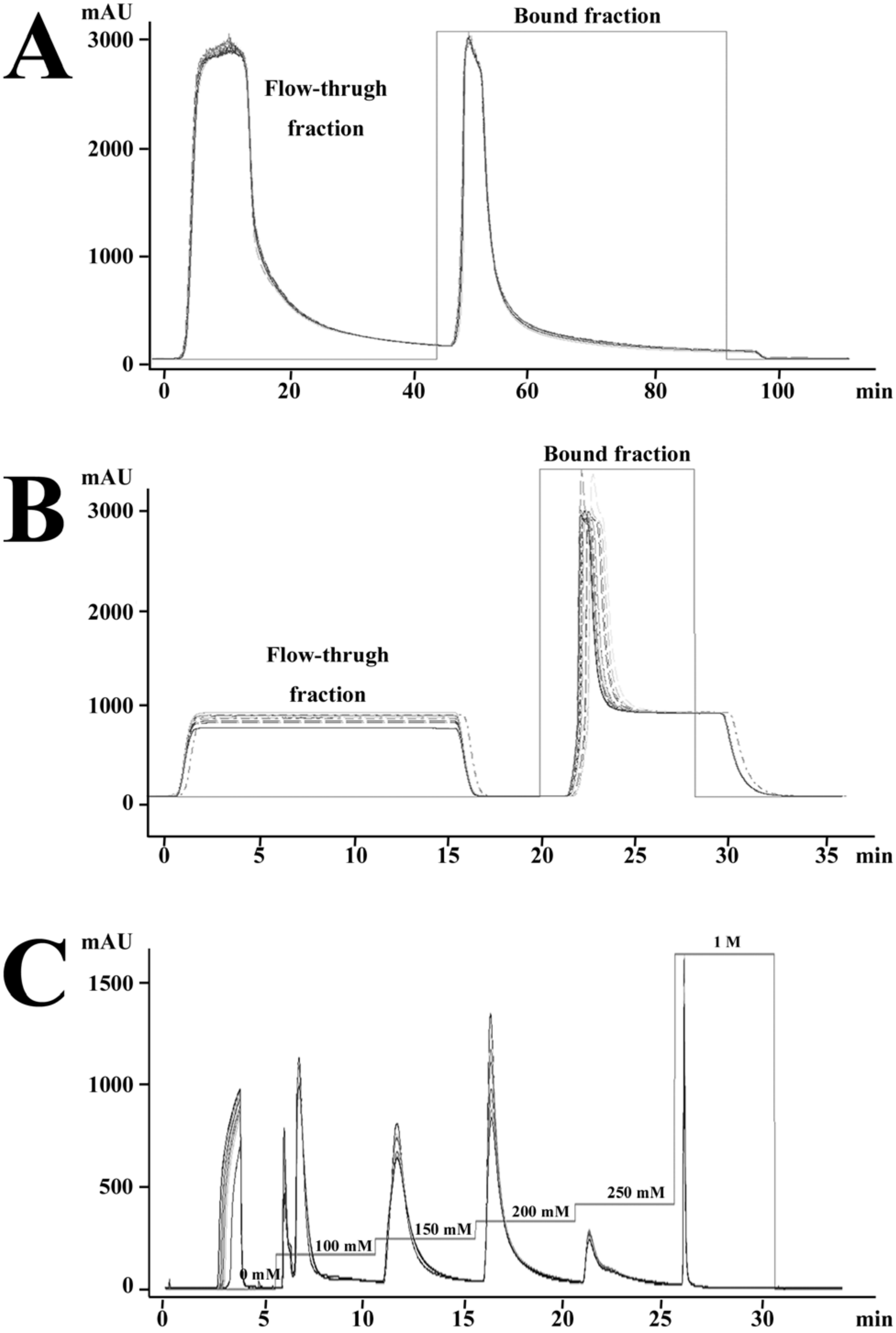

Supplementary figure 2

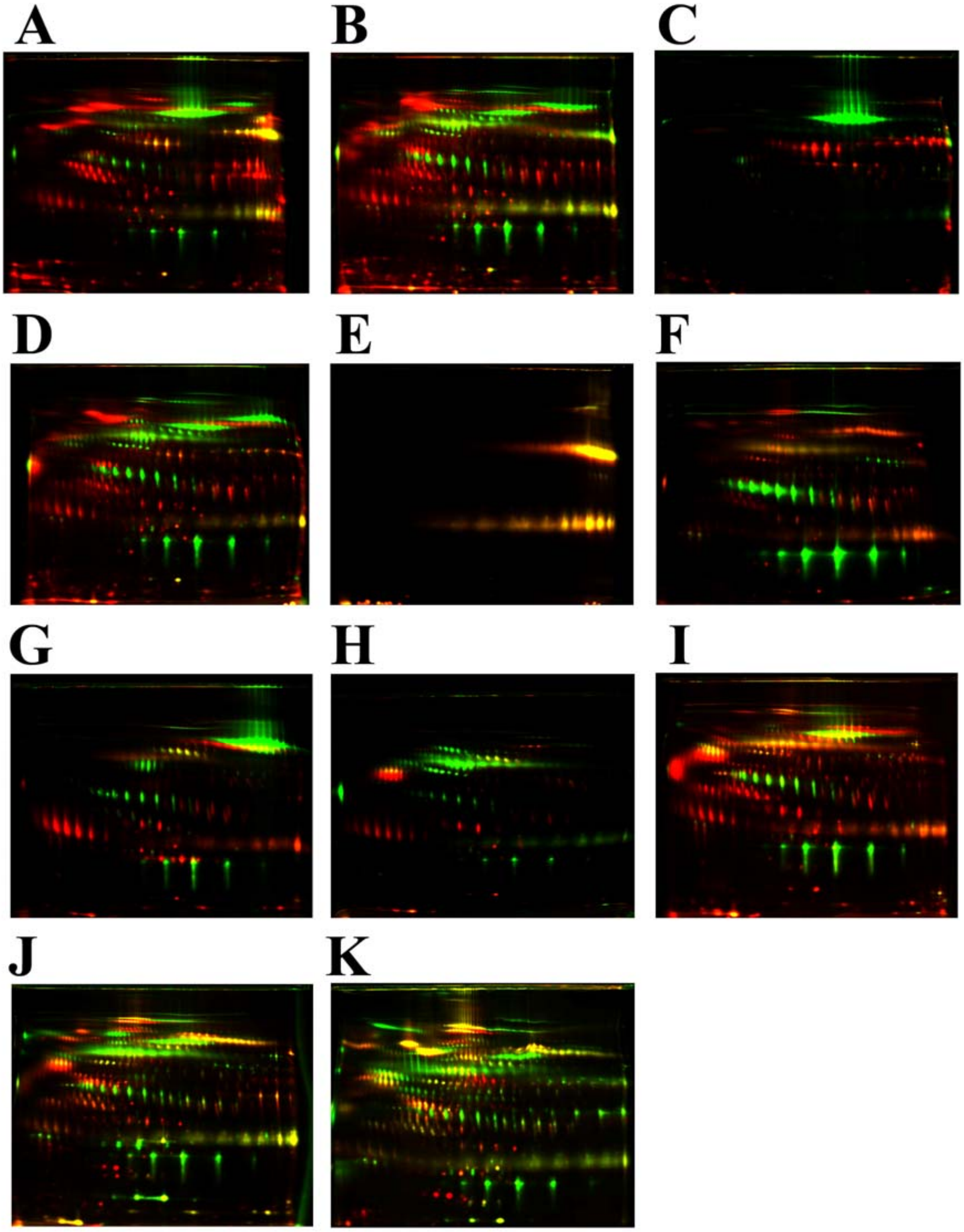

Supplement: Supplementary file 1 — Supplementary table 1: Recovery rate of protein samples after ProteoMiner treatment. Supplementary table 2: Number of protein spots from fractionated plasma samples. Supplementary table 3: Number of protein spots between the ProteoMiner-treated and untreated protein samples with different criteria. Supplementary table 4: Number of protein spots observed by mass spectrometric protein identification. Supplementary table 5: List of proteins identified by mass spectrometry. Supplementary table 6: Detailed data of identified proteins. Supplementary table 7: List of the identified proteins and their reported concentrations and references. Supplementary figure 1: Reproducibility of protein fractionation by liquid chromatography. The ultraviolet detection (280 nm) trace for each run demonstrated consistent separation and fractionation. A. HiTrap Blue HP column; B. HiTrap Protein G HP column; C. Resource Q column. Supplementary figure 2: Two-dimensional difference gel electrophoresis images of ProteoMiner-treated and untreated protein samples. The ProteoMiner-treated and untreated samples were labeled with Cy5 and Cy3, respectively. A. original plasma; B. flow-through fraction of HiTrap Blue HP column; C. binding fraction of HiTrap Blue HP column; D. flow-through fraction of HiTrap Protein G HP column; E. binding fraction of HiTrap Protein G HP column; 0 mM fraction (F), 100 mM fraction (G), 150 mM (H), 200 mM (I), 250 mM (J), and 1 M fraction (K) of Resource Q column. [file 739615.f1.pdf]
